# US Burden of Disorders Affecting the Nervous System: From the Global Burden of Disease 2021 Study

**DOI:** 10.1001/jamaneurol.2025.4470

**Published:** 2025-11-24

**Authors:** John P. Ney, Jaimie D. Steinmetz, Ellen Anderson-benge, Catherine W. Gillespie, Amanda Becker, Xaviera Steele, Gregory J. Esper

**Affiliations:** 1 Department of Neurology, Yale University, New Haven, Connecticut; 2 Institute for Health Metrics and Evaluation, University of Washington, Seattle; 3 American Academy of Neurology, Minneapolis, Minnesota; 4 Department of Neurology, Emory University, Atlanta, Georgia

## Abstract

**Importance:**

Nervous system health is a major contributor to population health, which is directly affected by neurological conditions and other disorders where nervous system damage occurs.

**Objective:**

To quantify aggregated health loss from diseases affecting the nervous system, including neurological disorders; neurodevelopmental disorders; congenital, neonatal, and systemic illnesses; and infectious diseases in the United States.

**Design, Setting, and Participants:**

This is a cross-sectional study of the Global Burden of Disease 2021 study data for nervous system health loss confined to the United States from 1990 through 2021 among the entire US population. Data analysis was performed from December 2021 to January 2025.

**Exposure:**

Thirty-six unique conditions that cause harm to the nervous system.

**Main Outcomes and Measures:**

Totals and age-standardized estimates with 95% uncertainty intervals (UIs) for disability-adjusted life-years (DALYs), years lived with disability (YLDs), years of life lost (YLLs), total attributable deaths (where applicable), and prevalence.

**Results:**

In 2021, of the US population of 332.7 million, disorders affecting nervous system health impacted 180.3 million (95% UI, 170.7 million to 190.4 million) US individuals and were the top cause of disability, with 16.6 million (95% UI, 12.9 million to 20.9 million) DALYs. The most prevalent conditions were tension-type headache (121.9 million; 95% UI, 109.4 million to 135.1 million), migraine (57.7 million; 95% UI, 50.1 million to 66.1 million), and diabetic neuropathy (17.1 million; 95% UI, 14.4 million to 19.9 million). Conditions with the greatest collective disability were stroke (3.9 million DALYs; 95% UI, 3.5 million to 4.2 million DALYs), Alzheimer disease and other dementias (3.3 million DALYs; 95% UI, 1.6 million to 6.9 million DALYs), diabetic neuropathy (2.2 million DALYs; 95% UI, 1.5 million to 3.0 million DALYs), and migraine (2.1 million DALYs; 95% UI, 0.4 million to 4.6 million DALYs). Compared with age-standardized metrics in 1990, the prevalence of disorders affecting the nervous system was nearly identical (−0.2%; 95% UI, −1.5% to 1.9%), with decreased attributable deaths (−14.6%; 95% UI, −18.3% to −11.3%) but increased YLDs (9.8%; 95% UI, 4.6% to 16.6%). By state, Mississippi, Alabama, and Louisiana had the largest age-standardized DALY rates, while New York, Massachusetts, and New Jersey had the smallest.

**Conclusions and Relevance:**

Disorders affecting the nervous system are highly prevalent and cause disability for millions of US individuals, with reduced mortality leading to more YLDS. The United States should prioritize efforts to combat these conditions with development and implementation of new and effective prevention strategies, therapeutics, and focused rehabilitation.

## Key Points

**Question:**

What is the US burden of disorders affecting the nervous system?

**Findings:**

This cross-sectional study of the Global Burden of Disease 2021 study data found that, among the US population of 332.7 million, disorders affecting nervous system health impacted 180.3 million US individuals and were the top cause of disability, with 16.6 million disability-adjusted life-years. Conditions with the greatest collective disability were stroke, Alzheimer disease and other dementias, diabetic neuropathy, and migraine.

**Meaning:**

Given the high prevalence of disorders affecting the nervous system, the United States should prioritize efforts to combat these conditions with new prevention strategies, therapeutics, and focused rehabilitation.

## Teaser

This cross-sectional study analyzes data from the Global Burden of Disease 2021 study to quantify aggregated health loss from diseases affecting the nervous system in the United States.

## Introduction

Nervous system health is intrinsic to human welfare. Healthy brain, spinal cord, and nerve function facilitates greater participation in employment^1^ and interpersonal relationships^2^ as well as improved quality of life.^3^ Disorders affecting the nervous system vary throughout the life course. Perinatal conditions affect infant neurological development,^4^ while neurodevelopmental conditions, epilepsy, migraine, and tension-type headache lead to demonstrable health loss in adolescents and working-age adults.^5,6,7^ Stroke and neurodegenerative diseases are prominent in older age,^7,8^ as are neurological implications of systemic disorders, such as diabetes.^9^

More than 1 in 3 persons worldwide has a condition impairing nervous system function, with net health loss of 443 million disability-adjusted life-years (DALYs) in 2021.^10^ Population growth and aging have accelerated the burden of neurological illness since 1990.^11,12^ However, prior analyses of population-level nervous system health in the United States have concentrated largely on age-related neurological diseases,^13,14^ discounting the contributions of congenital, neonatal, neurodevelopmental, and systemic illnesses that accrue from birth to older age. This article aims to summarize updated, comprehensive estimates of health losses associated with disorders affecting the nervous system in the United States using data from the Global Burden of Disease 2021 (GBD 2021) study as part of the Global Burden of Disease, Injuries, and Risk Factors study^15,16^ and is a product of the GBD Collaborator Network produced in accordance with the GBD protocol. The ongoing GBD study represents a collaborative effort to systematically quantify health loss by age, sex, location, and calendar year for all populations globally by synthesizing all available evidence and using modeling techniques and predictive covariates for estimates where data are lacking.

## Methods

The purpose of this secondary analysis generated as part of GBD 2021 was to isolate, aggregate, and summarize nervous system health loss across conditions modeled as part of the GBD study,^17^ using methods developed by Steinmetz et al,^10^ focusing on the United States. The University of Washington Institutional Review Board Committee approved the GBD study and determined that it was not human subjects research, rendering consents unnecessary. This study complies with the Guidelines on Accurate and Transparent Health Estimate Reporting (GATHER) recommendations.^18^ Data analysis was performed from December 2021 to January 2025.

Methods estimating condition-specific health loss due to disorders affecting the nervous system are detailed elsewhere.^19^ Briefly, GBD 2021 quantified health loss for 371 conditions in 204 countries and territories, including the United States, from 1990 through 2021.^17^ Steinmetz et al^10^ identified the subset of conditions impacting the nervous system to enable the most comprehensive estimate of nervous system health loss, including conditions where neurological health loss is the primary feature and conditions where neurological health loss is a consequence but not the primary feature. This set of conditions aligns with that of Steinmetz et al,^10^ with 1 exception. While malaria was included in the global analysis, it is not endemic in the United States and was excluded from the analysis. The remaining 36 conditions encompass a broad range of disorders affecting the nervous system: neurodevelopmental disorders, neurological disorders, and neurological consequences of other conditions, such as congenital, neonatal, metabolic, or infectious diseases (Table 1; eTables 1 and 2 in Supplement 1). The analysis includes estimates of neurological impacts of COVID-19 (defined in eTable 3 in Supplement 1).

**Table 1.** DALYs, Deaths, Prevalence, YLDs, and YLLs per 100 000 People and Age-Standardized Rates by Neurological Disorder Category, United States, 1990-2021

Cause and measure	No. in thousands (95% UI)		Age-standardized rate, No. (95% UI)/100 000 persons				
	In 2021	Change from 1990-2021, %	In 2021	Change from 1990-2021, %	Female	Male	Female to male ratio
**All neurological conditions**							
DALYs	16 563 (12 880 to 20 874)	55.2 (48.3 to 62.5)	3546.9 (2737.0 to 4584.8)	−4.3 (−7.2 to −1.2)	3532.5 (2563.9 to 4815.9)	3560.8 (2869.6 to 4353.3)	1.0 (0.9 to 1.1)
Deaths	479 (349 to 735)	64.2 (55.1 to 72.8)	75.8 (56.7 to 114.7)	−14.6 (−18.1 to −11.3)	73.5 (53.1 to 113.5)	77.6 (60.4 to 114.8)	0.9 (0.9 to 1.0)
Prevalence	180 334 (170 691 to 190 387)	35.4 (32.8 to 38.2)	49 980.6 (47 021.3 to 52 986.0)	0.2 (−1.5 to 1.9)	53 282.4 (50 301.1 to 56 282.7)	46 594.5 (43 500.5 to 49 922.2)	1.1 (1.1 to 1.2)
YLDs	9175 (6236 to 13 001)	67.5 (53.9 to 84.3)	2183.4 (1448.5 to 3183.7)	9.8 (4.6 to 16.6)	2260 (1366.1 to 3495.7)	2106.8 (1494.7 to 2900.8)	1.1 (0.9 to 1.3)
YLLs	7386 (5830 to 10 507)	42.5 (34.0 to 53.1)	1362.9 (1120.3 to 1829.5)	−20.3 (−23.6 to −16.5)	1272.1 (1012.0 to 1761.1)	1453.5 (1239.6 to 1898.4)	0.9 (0.8 to 0.9)
**Alzheimer disease and other dementias**							
DALYs	3319 (1552 to 6944)	85.4 (79.2 to 90.5)	509.7 (240.3 to 1065.7)	−3.6 (−5.6 to −1.9)	563.5 (269.9 to 1163.1)	437.3 (197.6 to 939.4)	1.3 (1.2 to 1.4)
Deaths	198 (51 to 491)	99.8 (90.2 to 108.8)	29.2 (7.8 to 73.5)	−0.9 (−4.2 to 1.8)	31.6 (8.6 to 77.9)	25.6 (6.6 to 67.1)	1.3 (1.2 to 1.3)
Prevalence	4877 (4232 to 5601)	78.2 (74.1 to 81.7)	773.1 (670.3 to 887.7)	−4.5 (−6.2 to −3.1)	855.2 (742.4 to 975.4)	668.3 (570.2 to 775.3)	1.3 (1.2 to 1.3)
YLDs	978 (673 to 1298)	74.3 (69.6 to 78.2)	154.1 (106.2 to 203.8)	−7.2 (−9.3 to −5.4)	175.8 (121.2 to 233.5)	125.9 (87.5 to 167.0)	1.4 (1.3 to 1.5)
YLLs	2340 (602 to 5919)	92.2 (83.4 to 100.4)	355.6 (90.9 to 896.1)	−1.3 (−4.6 to 1.4)	387.6 (100.6 to 952.8)	311.4 (77.4 to 814.2)	1.3 (1.2 to 1.3)
**Attention-deficit/hyperactivity disorder**							
DALYs	65 (35 to 109)	27.8 (17.5 to 38.5)	24.8 (13.1 to 41.7)	10.4 (0.9 to 20.0)	12.8 (7.0 to 21.0)	36.3 (19.1 to 62.5)	0.4 (0.3 to 0.4)
Prevalence	5362 (3880 to 7447)	28.3 (17.6 to 38.9)	2037.9 (1452.5 to 2883.8)	10.7 (1.0 to 20.0)	1059 (770.1 to 1486.6)	2984.4 (2099.9 to 4193.5)	0.4 (0.3 to 0.4)
YLDs	65 (35 to 109)	27.8 (17.5 to 38.5)	24.8 (13.1 to 41.7)	10.4 (0.9 to 20.0)	12.8 (7.0 to 21.0)	36.3 (19.1 to 62.5)	0.4 (0.3 to 0.4)
**Autism spectrum disorder**							
DALYs	639 (443 to 892)	28.9 (25.4 to 32.0)	203.3 (140.8 to 283.7)	1.8 (−0.9 to 4.1)	130.3 (88.6 to 182.1)	276.2 (192.0 to 386.0)	0.5 (0.4 to 0.5)
Prevalence	3491 (2920 to 4128)	30.9 (27.7 to 33.6)	1094.8 (920.2 to 1291.2)	2.4 (0.0 to 4.4)	706.2 (588.3 to 836.0)	1483.7 (1245.9 to 1748.4)	0.5 (0.4 to 0.5)
YLDs	639 (443 to 892)	28.9 (25.4 to 32.0)	203.3 (140.8 to 283.7)	1.8 (−0.9 to 4.1)	130.3 (88.6 to 182.1)	276.2 (192.0 to 386.0)	0.5 (0.4 to 0.5)
**Congenital birth defects**							
DALYs	21 (8 to 43)	30.5 (21.0 to 40.1)	6.6 (2.6 to 13.4)	2.4 (−4.5 to 9.8)	5.2 (1.8 to 11.0)	8 (3.2 to 15.9)	0.6 (0.4 to 0.8)
Prevalence	226 (103 to 401)	29.3 (21.1 to 37.0)	73.5 (36.7 to 126.4)	2.8 (−1.9 to 7.9)	56.3 (25.5 to 98.3)	90.8 (44.4 to 154.4)	0.6 (0.4 to 0.7)
YLDs	21 (8 to 43)	30.5 (21.0 to 40.1)	6.6 (2.6 to 13.4)	2.4 (−4.5 to 9.8)	5.2 (1.8 to 11.0)	8 (3.2 to 15.9)	0.6 (0.4 to 0.8)
**Congenital Zika syndrome**							
DALYs	0 (0 to 0)	100 (100.0 to 100.0)	0 (0.0 to 0.0)	100 (100.0 to 100.0)	0 (0.0 to 0.0)	0 (0.0 to 0.0)	1 (1.0 to 1.0)
Prevalence	0 (0 to 0)	100 (100.0 to 100.0)	0 (0.0 to 0.0)	100 (100.0 to 100.0)	0 (0.0 to 0.0)	0 (0.0 to 0.0)	1 (1.0 to 1.0)
YLDs	0 (0 to 0)	100 (100.0 to 100.0)	0 (0.0 to 0.0)	100 (100.0 to 100.0)	0 (0.0 to 0.0)	0 (0.0 to 0.0)	1 (1.0 to 1.0)
**COVID-19**							
DALYs	84 (3 to 262)	100 (100.0 to 100.0)	22.4 (0.8 to 77.9)	100 (100.0 to 100.0)	27.6 (0.8 to 90.8)	15.5 (0.7 to 58.5)	1.6 (0.8 to 2.4)
Prevalence	809 (159 to 2417)	100 (100.0 to 100.0)	220.2 (38.9 to 682.7)	100 (100.0 to 100.0)	280.8 (49.0 to 870.9)	158.1 (27.9 to 470.8)	1.8 (1.3 to 2.6)
YLDs	84 (3 to 262)	100 (100.0 to 100.0)	22.4 (0.8 to 77.9)	100 (100.0 to 100.0)	27.6 (0.8 to 90.8)	15.5 (0.7 to 58.5)	1.6 (0.8 to 2.4)
**Cystic echinococcosis**							
DALYs	0 (0 to 0)	22.1 (−4.0 to 51.5)	0 (0.0 to 0.0)	−12.7 (−31.1 to 7.5)	0 (0.0 to 0.0)	0 (0.0 to 0.0)	0.5 (0.4 to 0.6)
Prevalence	0 (0 to 0)	29.6 (20.2 to 41.0)	0 (0.0 to 0.0)	−7.3 (−13.0 to −0.0)	0 (0.0 to 0.0)	0 (0.0 to 0.1)	0.5 (0.4 to 0.6)
YLDs	0 (0 to 0)	22.1 (−4.0 to 51.5)	0 (0.0 to 0.0)	−12.7 (−31.1 to 7.5)	0 (0.0 to 0.0)	0 (0.0 to 0.0)	0.5 (0.4 to 0.6)
**Diabetes**							
DALYs	2183 (1481 to 2992)	359.1 (303.4 to 419.1)	392.3 (268.0 to 539.4)	151.8 (123.4 to 182.0)	336.8 (230.0 to 461.4)	455.8 (309.5 to 627.2)	0.7 (0.7 to 0.8)
Prevalence	17 060 (14 429 to 19 909)	367 (304.3 to 433.9)	3058.4 (2609.5 to 3539.3)	155.9 (124.4 to 188.8)	2679.7 (2286.6 to 3126.4)	3491 (2952.3 to 4084.4)	0.8 (0.7 to 0.8)
YLDs	2183 (1481 to 2992)	359.1 (303.4 to 419.1)	392.3 (268.0 to 539.4)	151.8 (123.4 to 182.0)	336.8 (230.0 to 461.4)	455.8 (309.5 to 627.2)	0.7 (0.7 to 0.8)
**Down syndrome**							
DALYs	9 (6 to 13)	15.7 (−5.6 to 42.1)	3.3 (2.2 to 4.7)	1.7 (−17.1 to 24.6)	3.1 (2.0 to 4.4)	3.5 (2.4 to 5.0)	0.9 (0.8 to 0.9)
Prevalence	99 (85 to 113)	13.9 (−6.8 to 40.0)	36.8 (31.8 to 42.4)	0.8 (−17.4 to 23.7)	34.4 (29.7 to 39.4)	39.2 (33.9 to 45.4)	0.9 (0.8 to 0.9)
YLDs	9 (6 to 13)	15.7 (−5.6 to 42.1)	3.3 (2.2 to 4.7)	1.7 (−17.1 to 24.6)	3.1 (2.0 to 4.4)	3.5 (2.4 to 5.0)	0.9 (0.8 to 0.9)
**Encephalitis**							
DALYs	27 (26 to 28)	83.5 (75.6 to 91.4)	8 (7.6 to 8.5)	35.2 (28.4 to 42.4)	7.1 (6.7 to 7.5)	9 (8.4 to 9.5)	0.8 (0.7 to 0.8)
Deaths	1 (1 to 1)	164.4 (150.3 to 175.1)	0.2 (0.2 to 0.2)	59.1 (52.1 to 65.9)	0.2 (0.2 to 0.2)	0.2 (0.2 to 0.2)	0.8 (0.7 to 0.8)
Prevalence	9 (6 to 12)	9.1 (1.5 to 16.1)	2.2 (1.6 to 2.9)	−27.6 (−32.3 to −23.2)	2.4 (1.7 to 3.0)	2.1 (1.5 to 2.7)	1.1 (1.1 to 1.2)
YLDs	1 (1 to 1)	7 (0.7 to 13.4)	0.2 (0.2 to 0.3)	−26.7 (−30.8 to −22.5)	0.3 (0.2 to 0.3)	0.2 (0.2 to 0.3)	1.2 (1.2 to 1.2)
YLLs	26 (25 to 27)	88.3 (79.9 to 95.7)	7.8 (7.4 to 8.2)	38.7 (31.6 to 46.2)	6.8 (6.4 to 7.3)	8.7 (8.2 to 9.3)	0.8 (0.7 to 0.8)
**Epilepsy**							
DALYs	415 (259 to 657)	51.1 (19.3 to 87.6)	117.8 (73.0 to 186.5)	10.5 (−12.2 to 36.6)	110.7 (67.4 to 176.8)	125 (79.0 to 195.9)	0.9 (0.8 to 0.9)
Deaths	3 (3 to 4)	99.7 (92.2 to 105.7)	0.8 (0.7 to 0.8)	29.3 (24.7 to 33.1)	0.7 (0.7 to 0.7)	0.9 (0.8 to 0.9)	0.8 (0.8 to 0.8)
Prevalence	1205 (771 to 1614)	57.9 (18.1 to 90.0)	341.6 (217.9 to 466.9)	14.1 (−13.5 to 37.7)	331.9 (211.4 to 456.2)	351.8 (224.7 to 479.0)	0.9 (0.9 to 1.0)
YLDs	295 (138 to 536)	47.2 (2.7 to 97.4)	83.8 (39.2 to 152.8)	6.7 (−23.8 to 42.6)	81.5 (38.4 to 147.5)	86.3 (39.7 to 156.7)	0.9 (0.9 to 1.0)
YLLs	120 (116 to 124)	61.9 (56.0 to 67.4)	34 (32.7 to 35.1)	21 (16.3 to 25.9)	29.3 (28.2 to 30.5)	38.7 (36.9 to 40.7)	0.8 (0.7 to 0.8)
**Fetal alcohol syndrome**							
DALYs	2 (1 to 4)	7.6 (−9.3 to 21.0)	0.8 (0.4 to 1.4)	−11.2 (−24.9 to −0.2)	0.7 (0.4 to 1.2)	0.9 (0.5 to 1.6)	0.8 (0.7 to 0.9)
Prevalence	74 (41 to 105)	16 (1.5 to 23.4)	24.5 (14.5 to 34.6)	−5.6 (−14.6 to 0.3)	21.7 (14.8 to 29.7)	27.2 (13.6 to 39.4)	0.8 (0.7 to 0.8)
YLDs	2 (1 to 4)	7.6 (−9.3 to 21.0)	0.8 (0.4 to 1.4)	−11.2 (−24.9 to −0.2)	0.7 (0.4 to 1.2)	0.9 (0.5 to 1.6)	0.8 (0.7 to 0.9)
**Guillain-Barré syndrome**							
DALYs	6 (4 to 8)	94.5 (67.3 to 126.1)	1.3 (0.8 to 1.8)	19.5 (5.2 to 36.2)	1.2 (0.8 to 1.7)	1.3 (0.9 to 1.9)	0.9 (0.9 to 1.0)
Prevalence	19 (17 to 23)	94.5 (67.3 to 126.1)	4.3 (3.7 to 5.0)	19.5 (5.2 to 36.2)	4.1 (3.6 to 4.8)	4.5 (3.9 to 5.2)	0.9 (0.9 to 1.0)
YLDs	6 (4 to 8)	94.5 (67.3 to 126.1)	1.3 (0.8 to 1.8)	19.5 (5.2 to 36.2)	1.2 (0.8 to 1.7)	1.3 (0.9 to 1.9)	0.9 (0.9 to 1.0)
**Idiopathic intellectual disability**							
DALYs	58 (17 to 109)	5.6 (−3.6 to 10.6)	19.5 (5.6 to 36.5)	−13.3 (−20.4 to −9.5)	16.8 (4.8 to 31.4)	22 (6.1 to 42.0)	NR
Prevalence	1151 (188 to 2184)	6.7 (−3.1 to 11.8)	386.6 (61.7 to 730.6)	−12.3 (−19.2 to −8.8)	339.9 (64.1 to 618.3)	431.4 (62.2 to 835.6)	NR
YLDs	58 (17 to 109)	5.6 (−3.6 to 10.6)	19.5 (5.6 to 36.5)	−13.3 (−20.4 to −9.5)	16.8 (4.8 to 31.4)	22 (6.1 to 42.0)	NR
**Klinefelter syndrome**							
DALYs	0 (0 to 0)	13 (1.6 to 25.2)	0 (0.0 to 0.1)	−2.1 (−12.5 to 8.6)	0 (0.0 to 0.0)	0.1 (0.0 to 0.1)	0 (0.0 to 0.0)
Prevalence	9 (6 to 13)	13.2 (8.6 to 17.5)	3.6 (2.3 to 5.1)	−1.6 (−4.2 to 1.5)	0 (0.0 to 0.0)	7 (4.6 to 10.0)	0 (0.0 to 0.0)
YLDs	0 (0 to 0)	13 (1.6 to 25.2)	0 (0.0 to 0.1)	−2.1 (−12.5 to 8.6)	0 (0.0 to 0.0)	0.1 (0.0 to 0.1)	0 (0.0 to 0.0)
**Meningitis**							
DALYs	36 (35 to 38)	−66.5 (−67.9 to −64.9)	11.7 (11.0 to 12.3)	−75.5 (−76.9 to −74.1)	10.3 (9.7 to 10.9)	13.1 (12.3 to 13.9)	0.8 (0.8 to 0.8)
Deaths	1 (1 to 1)	−47.7 (−49.9 to −45.4)	0.2 (0.2 to 0.2)	−68.6 (−69.9 to −67.2)	0.2 (0.2 to 0.2)	0.3 (0.3 to 0.3)	0.7 (0.7 to 0.8)
Prevalence	30 (24 to 41)	−69.6 (−72.5 to −66.7)	7.6 (6.0 to 10.2)	−79.8 (−81.6 to −77.9)	7.7 (6.0 to 10.3)	7.4 (5.8 to 10.2)	1 (1.0 to 1.1)
YLDs	2 (2 to 3)	−71.4 (−73.8 to −68.8)	0.6 (0.4 to 0.9)	−80.2 (−81.7 to −78.5)	0.7 (0.5 to 0.9)	0.6 (0.4 to 0.8)	1.1 (1.1 to 1.1)
YLLs	34 (33 to 36)	−66 (−67.5 to −64.5)	11 (10.4 to 11.7)	−75.2 (−76.7 to −73.7)	9.6 (9.1 to 10.2)	12.5 (11.7 to 13.3)	0.8 (0.7 to 0.8)
**Migraine**							
DALYs	2129 (351 to 4626)	26.3 (19.0 to 40.6)	614.7 (89.8 to 1356.5)	−3 (−7.5 to 1.4)	833.6 (106.3 to 1836.1)	394.9 (74.0 to 858.3)	2.1 (1.4 to 2.5)
Prevalence	57653 (50 085 to 66 079)	25.9 (20.4 to 31.4)	16 750.2 (14 529.8 to 19 303.4)	−2 (−5.8 to 1.9)	22 988.2 (19 967.9 to 26 374.0)	10 482.4 (8871.6 to 12 177.4)	2.2 (2.1 to 2.3)
YLDs	2129 (351 to 4626)	26.3 (19.0 to 40.6)	614.7 (89.8 to 1356.5)	−3 (−7.5 to 1.4)	833.6 (106.3 to 1836.1)	394.9 (74.0 to 858.3)	2.1 (1.4 to 2.5)
**Motor neuron disease**							
DALYs	211 (199 to 219)	88.6 (81.5 to 95.3)	41.4 (39.4 to 42.9)	4.1 (0.3 to 7.7)	33.5 (31.6 to 35.0)	50 (47.6 to 52.0)	0.7 (0.6 to 0.7)
Deaths	8 (8 to 9)	117.6 (109.0 to 126.0)	1.5 (1.4 to 1.6)	18.3 (13.9 to 22.7)	1.2 (1.1 to 1.3)	1.8 (1.7 to 1.9)	0.7 (0.6 to 0.7)
Prevalence	40 (38 to 44)	82.9 (66.3 to 99.7)	8.8 (8.3 to 9.5)	13.2 (3.1 to 23.7)	7.5 (7.0 to 8.1)	10.3 (9.7 to 11.1)	0.7 (0.7 to 0.7)
YLDs	9 (6 to 11)	82.8 (66.1 to 99.6)	1.9 (1.4 to 2.5)	13.2 (3.0 to 23.6)	1.6 (1.2 to 2.1)	2.2 (1.6 to 2.9)	0.7 (0.7 to 0.7)
YLLs	202 (191 to 210)	88.9 (81.4 to 95.6)	39.5 (37.8 to 41.0)	3.8 (−0.3 to 7.4)	31.9 (29.9 to 33.3)	47.8 (45.5 to 49.8)	0.7 (0.6 to 0.7)
**Multiple sclerosis**							
DALYs	219 (188 to 248)	80 (67.1 to 92.5)	48 (40.8 to 55.1)	11.5 (4.7 to 18.8)	62.9 (52.9 to 72.8)	32.2 (27.8 to 36.7)	2 (1.9 to 2.1)
Deaths	4 (4 to 5)	171.4 (153.1 to 190.7)	0.8 (0.8 to 0.9)	43.9 (34.5 to 53.6)	1 (0.9 to 1.0)	0.6 (0.6 to 0.6)	1.6 (1.5 to 1.7)
Prevalence	419 (386 to 453)	54.6 (39.9 to 69.8)	100 (92.1 to 108.3)	5.4 (−4.4 to 15.5)	141.1 (130.2 to 153.0)	57.1 (52.6 to 62.4)	2.5 (2.4 to 2.6)
YLDs	103 (74 to 131)	51.7 (35.9 to 67.5)	24.9 (17.9 to 31.9)	4.3 (−5.9 to 15.1)	34.8 (25.0 to 44.7)	14.5 (10.4 to 18.7)	2.4 (2.3 to 2.5)
YLLs	116 (109 to 122)	115.9 (102.5 to 129.8)	23.1 (21.9 to 24.2)	20.8 (13.9 to 28.5)	28 (26.2 to 29.7)	17.7 (16.7 to 18.7)	1.6 (1.5 to 1.7)
**Neonatal encephalopathy**							
DALYs	225 (194 to 257)	−23.6 (−34.9 to −12.9)	103.9 (91.3 to 116.0)	−25.9 (−36.0 to −16.9)	92.5 (81.9 to 103.2)	114.9 (99.8 to 129.6)	0.8 (0.8 to 0.9)
Deaths	1 (1 to 2)	−41.2 (−47.8 to −33.9)	0.8 (0.7 to 0.9)	−33.7 (−40.9 to −25.4)	0.7 (0.7 to 0.8)	0.8 (0.7 to 0.9)	0.9 (0.8 to 1.0)
Prevalence	492 (426 to 559)	23.8 (−9.5 to 50.4)	156.9 (136.3 to 178.3)	−2.5 (−28.9 to 18.7)	118.6 (102.9 to 135.0)	195 (169.4 to 222.0)	0.6 (0.6 to 0.6)
YLDs	99 (72 to 131)	28.1 (−27.2 to 80.0)	32.8 (23.9 to 43.4)	2.7 (−41.6 to 43.4)	25.1 (18.3 to 33.1)	40.5 (29.2 to 54.1)	0.6 (0.6 to 0.7)
YLLs	126 (112 to 140)	−41.2 (−47.8 to −33.8)	71 (63.1 to 79.2)	−33.7 (−40.8 to −25.4)	67.4 (60.3 to 75.3)	74.4 (65.8 to 84.6)	0.9 (0.8 to 1.0)
**Neonatal jaundice**							
DALYs	6 (4 to 8)	32.8 (25.9 to 41.3)	2 (1.5 to 2.8)	6.3 (0.7 to 13.2)	2 (1.4 to 2.7)	2.1 (1.5 to 2.8)	0.9 (0.9 to 1.0)
Prevalence	17 (15 to 19)	31.9 (27.4 to 36.1)	5.6 (5.0 to 6.3)	6 (2.5 to 9.4)	5.4 (4.9 to 6.1)	5.7 (5.2 to 6.4)	0.9 (0.9 to 1.0)
YLDs	6 (4 to 8)	32.8 (25.9 to 41.3)	2 (1.5 to 2.8)	6.3 (0.7 to 13.2)	2 (1.4 to 2.7)	2.1 (1.5 to 2.8)	0.9 (0.9 to 1.0)
**Neonatal sepsis**							
DALYs	31 (18 to 45)	9.1 (−31.6 to 74.3)	10.1 (6.1 to 14.8)	−12.7 (−45.2 to 40.0)	9.3 (5.6 to 13.6)	10.9 (6.5 to 16.0)	0.9 (0.8 to 0.9)
Prevalence	83 (60 to 109)	6.4 (−30.6 to 63.8)	27.5 (19.7 to 36.1)	−14.5 (−44.1 to 31.2)	25.3 (18.1 to 33.1)	29.6 (21.3 to 38.9)	0.9 (0.8 to 0.9)
YLDs	31 (18 to 45)	9.1 (−31.6 to 74.3)	10.1 (6.1 to 14.8)	−12.7 (−45.2 to 40.0)	9.3 (5.6 to 13.6)	10.9 (6.5 to 16.0)	0.9 (0.8 to 0.9)
**Nervous system cancer**							
DALYs	610 (585 to 626)	35.1 (31.1 to 38.3)	139.9 (135.3 to 143.9)	−16.1 (−18.3 to −13.9)	114.8 (110.4 to 118.8)	166.8 (160.7 to 171.8)	0.7 (0.7 to 0.7)
Deaths	22 (20 to 23)	61.2 (55.8 to 65.5)	4.2 (3.9 to 4.3)	−8.6 (−11.4 to −6.4)	3.4 (3.2 to 3.5)	5.1 (4.8 to 5.2)	0.7 (0.6 to 0.7)
Prevalence	88 (84 to 91)	46.1 (40.6 to 50.9)	24.9 (23.8 to 26.0)	5.1 (0.8 to 9.3)	22.8 (21.6 to 24.0)	27.2 (25.8 to 28.5)	0.8 (0.8 to 0.9)
YLDs	11 (8 to 15)	54.4 (47.8 to 60.6)	2.8 (2.0 to 3.6)	0.4 (−3.7 to 4.7)	2.4 (1.8 to 3.1)	3.1 (2.3 to 4.1)	0.8 (0.7 to 0.8)
YLLs	598 (575 to 614)	34.8 (31.1 to 37.9)	137.2 (132.7 to 140.8)	−16.4 (−18.5 to −14.2)	112.4 (108.1 to 116.2)	163.7 (157.8 to 169.0)	0.7 (0.7 to 0.7)
**Neural tube defects**							
DALYs	45 (38 to 51)	−39.5 (−52.0 to −28.6)	21.5 (17.9 to 24.7)	−40.9 (−54.4 to −29.6)	24.1 (20.2 to 27.7)	19 (15.1 to 22.2)	1.3 (1.1 to 1.5)
Deaths	0 (0 to 1)	−43.5 (−55.5 to −33.8)	0.2 (0.2 to 0.3)	−43.8 (−57.1 to −32.9)	0.3 (0.2 to 0.3)	0.2 (0.2 to 0.2)	1.3 (1.1 to 1.5)
Prevalence	29 (24 to 35)	123.4 (84.7 to 161.4)	9.3 (7.8 to 11.0)	66.2 (37.0 to 95.0)	10 (8.4 to 11.8)	8.6 (7.2 to 10.2)	1.2 (1.1 to 1.2)
YLDs	8 (6 to 12)	120.6 (82.0 to 159.5)	2.7 (1.8 to 3.8)	65.4 (36.4 to 94.8)	2.9 (2.0 to 4.0)	2.5 (1.7 to 3.5)	1.2 (1.1 to 1.2)
YLLs	37 (30 to 42)	−48 (−60.4 to −38.5)	18.8 (15.1 to 21.8)	−45.8 (−59.4 to −34.7)	21.2 (16.9 to 24.6)	16.5 (12.4 to 19.7)	1.3 (1.1 to 1.6)
**Neurocysticercosis**							
DALYs	92 (53 to 150)	77.9 (41.3 to 123.4)	18.8 (10.4 to 30.1)	7.2 (−14.0 to 32.3)	20.6 (11.3 to 33.1)	16.9 (9.3 to 28.1)	1.2 (1.1 to 1.3)
Deaths	0 (0 to 0)	−38.9 (−72.8 to 20.2)	0 (0.0 to 0.0)	−60.2 (−82.3 to −22.3)	0 (0.0 to 0.0)	0 (0.0 to 0.0)	0.9 (0.2 to 2.8)
Prevalence	456 (326 to 607)	95.7 (73.2 to 122.4)	91.6 (64.4 to 121.6)	16.9 (7.6 to 26.6)	100.7 (72.1 to 132.3)	81.7 (56.6 to 111.9)	1.2 (1.1 to 1.3)
YLDs	92 (53 to 150)	78.4 (41.6 to 124.1)	18.8 (10.4 to 30.0)	7.5 (−13.6 to 33.0)	20.6 (11.2 to 33.0)	16.9 (9.3 to 28.1)	1.2 (1.1 to 1.3)
YLLs	0 (0 to 0)	−42.7 (−74.8 to 14.9)	0 (0.0 to 0.0)	−58.9 (−83.1 to −15.0)	0 (0.0 to 0.0)	0 (0.0 to 0.1)	0.9 (0.2 to 2.8)
**Neurosyphilis**							
DALYs	0 (0 to 1)	21 (6.3 to 39.2)	0.1 (0.0 to 0.1)	−24.7 (−34.4 to −14.3)	0 (0.0 to 0.0)	0.1 (0.1 to 0.2)	0.2 (0.1 to 0.3)
Prevalence	6 (5 to 8)	18.3 (2.4 to 35.4)	1.7 (1.2 to 2.2)	−16.4 (−27.5 to −4.2)	1.4 (1.0 to 1.9)	1.9 (1.5 to 2.5)	0.7 (0.6 to 0.8)
YLDs	0 (0 to 1)	21 (6.3 to 39.2)	0.1 (0.0 to 0.1)	−24.7 (−34.4 to −14.3)	0 (0.0 to 0.0)	0.1 (0.1 to 0.2)	0.2 (0.1 to 0.3)
**Other chromosomal anomalies**							
DALYs	16 (11 to 23)	11.3 (−1.3 to 24.3)	6.3 (4.2 to 9.0)	−0.9 (−11.9 to 10.5)	6.2 (4.1 to 8.6)	6.4 (4.2 to 9.5)	1 (0.8 to 1.2)
Prevalence	180 (162 to 203)	9.9 (−1.9 to 22.8)	70.6 (63.1 to 79.8)	−1.1 (−11.6 to 10.3)	69 (60.6 to 79.5)	72 (60.3 to 85.1)	1 (0.8 to 1.2)
YLDs	16 (11 to 23)	11.3 (−1.3 to 24.3)	6.3 (4.2 to 9.0)	−0.9 (−11.9 to 10.5)	6.2 (4.1 to 8.6)	6.4 (4.2 to 9.5)	1 (0.8 to 1.2)
**Other neurological disorders**							
DALYs	332 (297 to 373)	81.6 (71.6 to 93.0)	80.1 (70.2 to 93.5)	13.8 (6.0 to 24.6)	70.6 (60.5 to 84.3)	90.2 (80.4 to 103.5)	0.8 (0.7 to 0.8)
Deaths	10 (9 to 10)	127.7 (113.7 to 135.5)	1.8 (1.7 to 1.9)	25.1 (17.5 to 28.8)	1.5 (1.3 to 1.6)	2.2 (2.1 to 2.3)	0.7 (0.6 to 0.7)
YLLs	237 (221 to 246)	64.1 (54.5 to 69.2)	54.5 (51.3 to 56.3)	−2.3 (−7.6 to 0.6)	43.3 (39.0 to 45.1)	66.3 (63.7 to 68.6)	0.7 (0.6 to 0.7)
**Parkinson disease**							
DALYs	613 (547 to 657)	163.5 (153.4 to 171.2)	97.7 (87.4 to 104.7)	43.5 (38.3 to 47.5)	64.6 (56.1 to 70.3)	141.2 (128.4 to 149.9)	0.5 (0.4 to 0.5)
Deaths	37 (32 to 40)	183.4 (173.8 to 190.5)	5.8 (5.0 to 6.3)	52.9 (49.0 to 56.2)	3.8 (3.1 to 4.2)	8.7 (7.7 to 9.2)	0.4 (0.4 to 0.5)
Prevalence	692 (636 to 752)	137.8 (107.7 to 170.2)	111.9 (102.8 to 121.5)	29.4 (13.3 to 46.3)	81.7 (75.1 to 88.6)	149.5 (137.5 to 162.4)	0.5 (0.5 to 0.6)
YLDs	94 (69 to 125)	131 (101.5 to 160.9)	15.3 (11.2 to 20.2)	25.7 (10.0 to 41.3)	11.2 (8.1 to 14.7)	20.5 (14.9 to 27.1)	0.5 (0.5 to 0.6)
YLLs	519 (456 to 550)	170.6 (162.6 to 177.1)	82.3 (73.1 to 87.2)	47.5 (43.8 to 50.7)	53.5 (45.4 to 57.6)	120.8 (109.2 to 126.8)	0.4 (0.4 to 0.5)
**Preterm birth**							
DALYs	443 (316 to 574)	16.2 (7.0 to 26.0)	151.4 (107.6 to 197.3)	−4.2 (−12.0 to 4.1)	152.5 (108.4 to 198.1)	150.1 (106.3 to 195.0)	1 (1.0 to 1.0)
Prevalence	3942 (3210 to 4647)	28.1 (18.7 to 38.2)	1236.5 (1014.1 to 1449.7)	0.2 (−6.9 to 7.9)	1215.5 (1001.1 to 1419.7)	1256.8 (1027.5 to 1477.6)	1 (1.0 to 1.0)
YLDs	443 (316 to 574)	16.2 (7.0 to 26.0)	151.4 (107.6 to 197.3)	−4.2 (−12.0 to 4.1)	152.5 (108.4 to 198.1)	150.1 (106.3 to 195.0)	1 (1.0 to 1.0)
**Rabies**							
DALYs	0 (0 to 0)	187.8 (155.9 to 221.8)	0.1 (0.1 to 0.1)	157.5 (127.3 to 190.8)	0.1 (0.1 to 0.1)	0.1 (0.1 to 0.1)	1 (0.9 to 1.1)
Deaths	0 (0 to 0)	297.2 (255.1 to 337.2)	0 (0.0 to 0.0)	178.9 (149.0 to 210.5)	0 (0.0 to 0.0)	0 (0.0 to 0.0)	1 (0.8 to 1.0)
Prevalence	0 (0 to 0)	297.2 (252.2 to 339.0)	0 (0.0 to 0.0)	178.9 (149.3 to 209.9)	0 (0.0 to 0.0)	0 (0.0 to 0.0)	1 (0.8 to 1.0)
YLDs	0 (0 to 0)	297.2 (252.2 to 339.0)	0 (0.0 to 0.0)	178.9 (149.3 to 209.9)	0 (0.0 to 0.0)	0 (0.0 to 0.0)	1 (0.8 to 1.0)
YLLs	0 (0 to 0)	187.8 (156.3 to 221.7)	0.1 (0.1 to 0.1)	157.5 (127.6 to 191.0)	0.1 (0.1 to 0.1)	0.1 (0.1 to 0.1)	1 (0.9 to 1.1)
**Spinal cord injury**							
DALYs	310 (218 to 400)	11.1 (4.3 to 17.8)	72.7 (50.6 to 93.7)	−27.7 (−31.1 to −24.2)	51.8 (36.5 to 66.8)	94.2 (65.2 to 121.8)	0.6 (0.5 to 0.6)
Prevalence	1173 (1075 to 1276)	15.3 (9.4 to 22.4)	270.6 (249.9 to 294.1)	−25.8 (−28.9 to −22.4)	200.8 (182.4 to 219.4)	342.4 (315.3 to 372.1)	0.6 (0.6 to 0.6)
YLDs	310 (218 to 400)	11.1 (4.3 to 17.8)	72.7 (50.6 to 93.7)	−27.7 (−31.1 to −24.2)	51.8 (36.5 to 66.8)	94.2 (65.2 to 121.8)	0.6 (0.5 to 0.6)
**Stroke**							
DALYs	3912 (3533 to 4227)	28 (23.3 to 31.6)	694.9 (636.0 to 750.1)	−27.9 (−30.3 to −25.9)	650.7 (584.4 to 709.9)	739.3 (686.6 to 793.6)	0.9 (0.8 to 0.9)
Deaths	192 (163 to 207)	30.7 (24.4 to 35.2)	30.3 (26.0 to 32.5)	−31.3 (−34.2 to −29.1)	28.9 (24.0 to 31.6)	31.2 (28.1 to 33.1)	0.9 (0.9 to 1.0)
Prevalence	6299 (5889 to 6761)	67.3 (60.1 to 74.3)	1207 (1130.8 to 1293.3)	−1.6 (−5.5 to 2.2)	1160.6 (1089.4 to 1242.4)	1264.3 (1177.9 to 1358.1)	0.9 (0.9 to 0.9)
YLDs	882 (641 to 1123)	64.5 (56.9 to 72.1)	166.9 (121.0 to 211.8)	−3.6 (−7.8 to 0.5)	169.9 (124.0 to 216.5)	164.5 (118.4 to 209.0)	1 (1.0 to 1.1)
YLLs	3030 (2709 to 3212)	20.3 (14.7 to 24.5)	528 (478.6 to 558.0)	−33.3 (−35.9 to −30.9)	480.8 (423.3 to 512.5)	574.8 (534.2 to 605.1)	0.8 (0.8 to 0.9)
**Tension-type headache**							
DALYs	261 (72 to 916)	36.9 (23.1 to 46.7)	70.3 (18.0 to 258.3)	−1.5 (−6.7 to 2.1)	78.5 (21.0 to 278.7)	61.9 (15.1 to 232.3)	1.4 (0.9 to 1.5)
Prevalence	121 925 (109 446 to 135 070)	29 (24.3 to 33.8)	34 320.6 (30 762.9 to 38 348.9)	−2.7 (−5.6 to 0.3)	36 004.9 (32 285.9 to 40 181.0)	32 608.8 (28 914.3 to 36 554.8)	1.1 (1.1 to 1.2)
YLDs	261 (72 to 916)	36.9 (23.1 to 46.7)	70.3 (18.0 to 258.3)	−1.5 (−6.7 to 2.1)	78.5 (21.0 to 278.7)	61.9 (15.1 to 232.3)	1.4 (0.9 to 1.5)
**Tetanus**							
DALYs	0 (0 to 0)	−78.8 (−83.3 to −73.9)	0 (0.0 to 0.0)	−86.2 (−88.8 to −83.2)	0 (0.0 to 0.0)	0 (0.0 to 0.0)	1 (0.7 to 1.5)
Deaths	0 (0 to 0)	−75.5 (−80.8 to −69.8)	0 (0.0 to 0.0)	−86.1 (−89.0 to −82.9)	0 (0.0 to 0.0)	0 (0.0 to 0.0)	1 (0.7 to 1.6)
Prevalence	0 (0 to 0)	−26.9 (−43.6 to −10.5)	0 (0.0 to 0.0)	−42.4 (−55.9 to −29.3)	0 (0.0 to 0.0)	0 (0.0 to 0.0)	0.8 (0.7 to 0.9)
YLDs	0 (0 to 0)	−49.2 (−65.4 to −30.6)	0 (0.0 to 0.0)	−59.7 (−73.5 to −44.1)	0 (0.0 to 0.0)	0 (0.0 to 0.0)	0.9 (0.8 to 1.0)
YLLs	0 (0 to 0)	−78.9 (−83.6 to −73.8)	0 (0.0 to 0.0)	−86.3 (−89.0 to −83.3)	0 (0.0 to 0.0)	0 (0.0 to 0.0)	1 (0.7 to 1.5)
**Traumatic brain injury**							
DALYs	242 (171 to 323)	26.7 (22.4 to 31.6)	51.4 (36.1 to 68.3)	−23.2 (−25.4 to −20.8)	38.1 (27.0 to 51.1)	65.5 (45.8 to 87.3)	0.6 (0.6 to 0.6)
Prevalence	1874 (1779 to 1973)	29.2 (25.3 to 33.6)	393 (373.5 to 412.3)	−22.3 (−24.2 to −20.1)	296.1 (278.9 to 313.9)	495.6 (473.0 to 520.4)	0.6 (0.6 to 0.6)
YLDs	242 (171 to 323)	26.7 (22.4 to 31.6)	51.4 (36.1 to 68.3)	−23.2 (−25.4 to −20.8)	38.1 (27.0 to 51.1)	65.5 (45.8 to 87.3)	0.6 (0.6 to 0.6)

Abbreviations: DALYs, disability-adjusted life-years; NR, not reported; UI, uncertainty interval; YLDs, years lived with disability; YLLs, years of life lost.

Reference case definitions, the number of data sources for models, and data collection methods were identified for each condition as part of the GBD study (eTables 3-6 in Supplement 1). The condition-specific measures of health loss quantified in GBD that were summarized in the present analysis were prevalence, deaths, and the standard burden metrics reported by the GBD study: years lived with disability (YLDs), years of life lost (YLLs) due to premature mortality, and DALYs.^15,16,19^ GBD estimation methods and input sources vary by measure of health loss and condition. All condition-specific input sources are searchable in the Global Health Data Exchange (https://ghdx.healthdata.org/gbd-2021/sources).^20^

For most conditions, prevalence by age, sex, location, and calendar year was estimated using bayesian models informed by population-representative studies, large-scale surveys, censuses, insurance claims, hospital records, and predictive covariates. Condition-specific prevalence estimates were stratified by subtype or severity level, termed *sequalae*, each associated with disability weight, enabling estimation of YLDs: the product of sequelae-specific prevalence and disability weight.

For 15 conditions that could result in death directly from neurological damage (eTable 1 in Supplement 1), mortality estimates by age, sex, location, and calendar year were modeled using diagnostic-coded (eg, *International Classification of Diseases*) vital registration data, along with registry, mortuary, hospital, police, and census data, correcting for nonspecific diagnoses and systemic undercoding. Age-specific mortality estimates translate to YLLs by multiplying age-specific deaths by remaining standard life expectancy.

Last, DALYs were estimated by summing YLDs and YLLs for each condition, age, sex, location, and calendar year. DALYs equal YLDs for nonfatal conditions. Deaths and YLLs are excluded for conditions leading to death through other mechanisms (eg, fatality in acute respiratory COVID-19 distinct from long-term cognitive impairment).

### Statistical Analysis

Aggregate measures of health loss due to all 36 conditions were generated leveraging a comorbidity correction under assumption of independence between conditions. We estimated the total population with any disorder affecting the nervous system in the United States as follows: total prevalence = 1 – [(1 – prevalence of condition 1) × (1 – prevalence of condition 2) × … (1 – prevalence of condition 36)]. Estimates were compiled using DisMod-MR 2.1 (Institute for Health Metrics and Evaluation). All estimates are accompanied by 95% uncertainty intervals (UIs) reflecting 2.5th and 97.5th percentile values from 500 ordered draws. We were not testing hypotheses, so significance and *P* values were not included. We present raw counts, rates distributed among age categories, age-standardized rates calculated using standard GBD age weights, temporal changes in health metrics (calculated by subtracting 1990 estimates from 2021 estimates and dividing the difference by 1990 estimates), and sex ratios (calculated by dividing age-standardized rates for females by age-standardized rates for males), as well as age-adjusted DALY rates by state.

## Results

### Raw Counts and Trends

**Figure 1.**
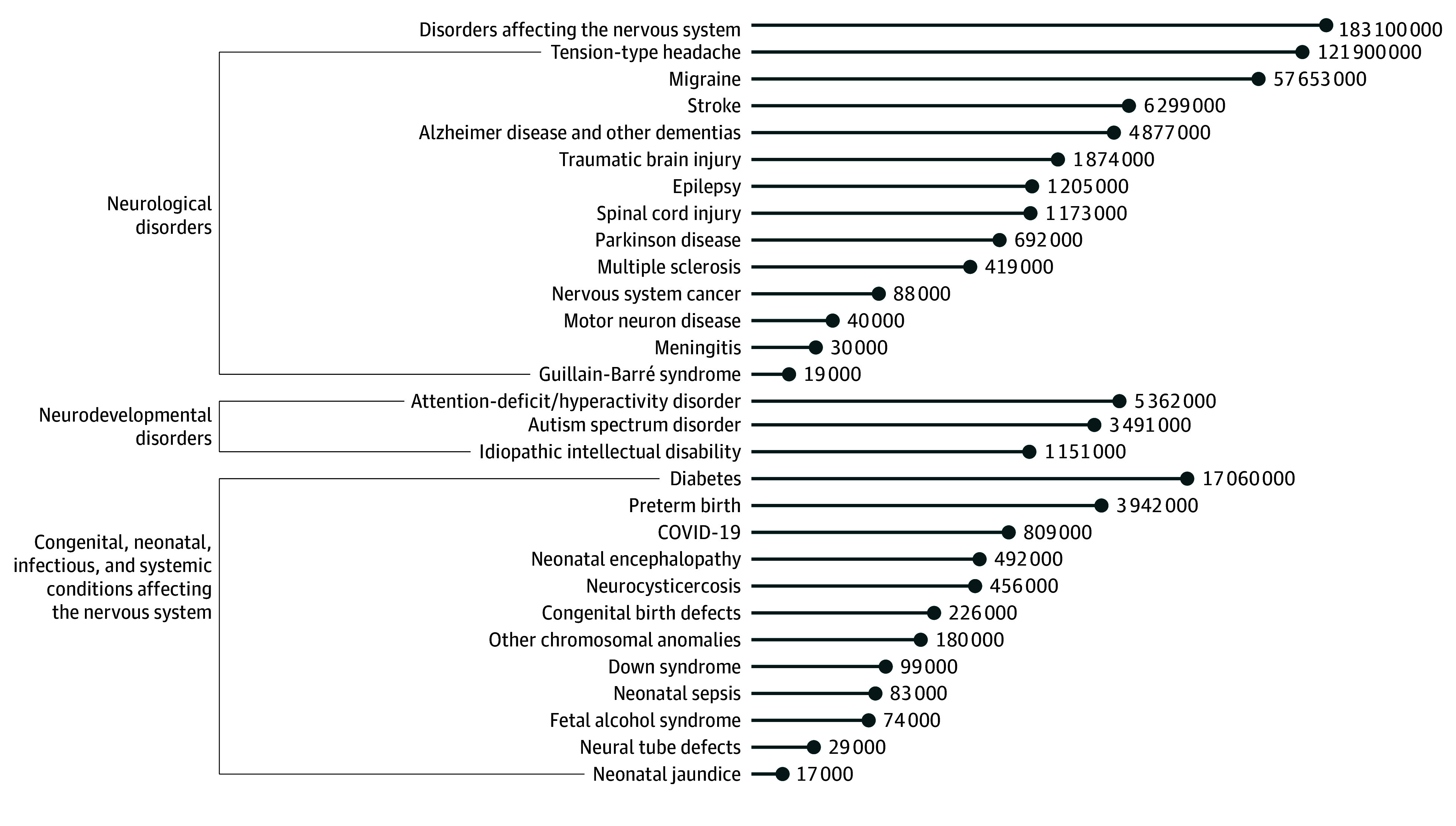
Estimated Prevalence of Conditions Affecting the Nervous System in the United States in 2021 by Category Numbers indicate the number of US individuals affected. Conditions with fewer than 1 million cases are not depicted. Conditions may overlap and will exceed the estimated total prevalence.

**Figure 2.**
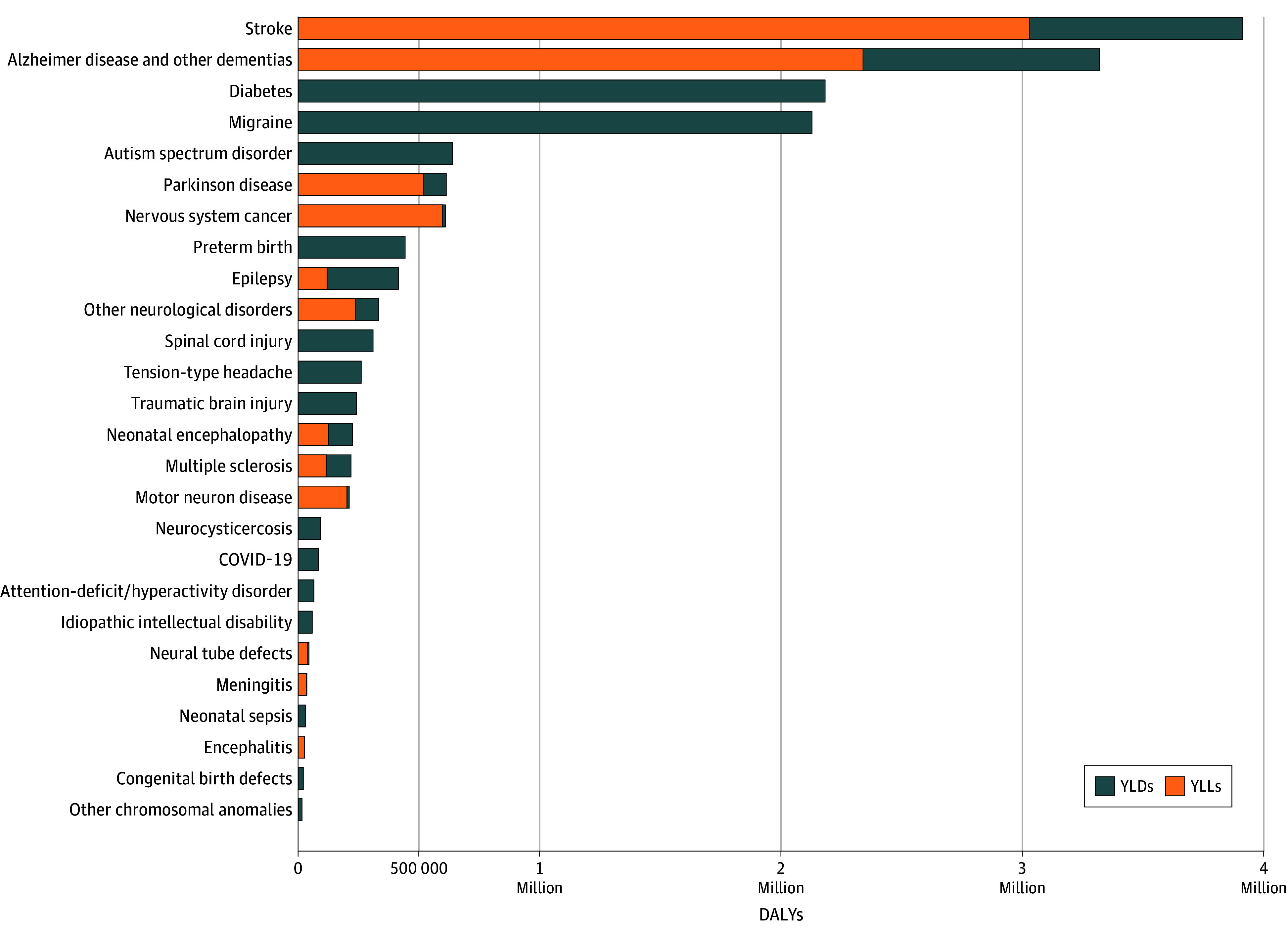
Conditions Affecting Nervous System Health by Disability-Adjusted Life-Years (DALYs) in the United States in 2021, by Relative Contribution to Total DALYs There were a total of 16.6 million total DALYs for disorders affecting the nervous system in 2021. A category of aggregated neurological conditions described in Table 1 as “other neurological disorders” is included. Years lived with disability (YLDs) and years of life lost (YLLs) were summed by condition to yield DALYs. Where deaths are not associated with the condition (eg, migraine), no YLLs were calculated (see Methods). Values less than 100 000 DALYs are not depicted.

In 2021, 54.2% of US individuals (180.3 million; 95% UI, 170.7 million to 190.4 million) had at least 1 of 36 unique medical conditions impacting nervous system health (Figure 1). Tension-type headache was the most prevalent condition (121.9 million; 95% UI, 109.4 million to 135.1 million) followed by migraine, diabetic neuropathy, and stroke. There were 479 000 (95% UI, 349 000 to 735 000) attributable deaths, with the greatest number from Alzheimer disease and other dementias (198 000; 95% UI, 51 200 to 491 000), followed by stroke, Parkinson disease, and nervous system cancers. Disorders affecting the nervous system led to 7.4 million YLLs (95% UI, 5.8 million to 10.5 million YLLs), 9.2 million YLDs (95% UI, 6.2 million to 13.0 million YLDs), and 16.6 million DALYs (95% UI, 12.9 million to 20.9 million DALYs). Stroke had the greatest net health loss (3.9 million DALYs; 95% UI, 3.5 million to 4.2 million DALYs), then Alzheimer disease and other dementias, diabetic neuropathy, and migraine. Diabetic neuropathy led to the most nonfatal disability (2.2 million YLDs; 95% UI, 1.5 million to 3.0 million YLDs), then migraine, Alzheimer disease and other dementias, and stroke (Figure 2). Since 1990, the prevalence of disorders affecting neurological health increased by 34.4% (95% UI, 31.7% to 37.4%), DALYs increased by 55.2% (95% UI, 48.3% to 62.5%), deaths due to these conditions rose 64.2% (95% UI, 55.1% to 72.8%), and YLDs and YLLs grew 67.5% (95% UI, 53.9% to 84.3%) and 42.5% (95% UI, 34.0% to 53.1%), respectively (Table 1).

### Rates by Age Category

Individuals in the United States aged 80 years or older had the greatest rates of nervous system health loss at 32 928.0 DALYs/100 000 individuals (95% UI, 24 321.8 to 48 158.1 DALYs/100 000 individuals). Neurological health loss for those younger than 5 years was 1739.4 DALYs/100 000 individuals (95% UI, 1552.7 to 1933.9 DALYs/100 000 individuals), which was greater than that for individuals aged 5 to 19 years, with 2-fold to 4-fold increases in DALY rates in each subsequent age category (20-59, 60-79, and ≥80 years).

### Age-Standardized Rates

The age-standardized prevalence rate of these disorders in 2021 was 49 981 per 100 000 individuals (95% UI, 47 021 to 52 986 individuals), a 0.2% (95% UI, −1.5% to 1.9%) gain compared with the rate in 1990. Age-adjusted rates in 2021 declined 4.3% (95% UI, 1.2% to 7.2%) for DALYs, 14.6% (95% UI, 11.3% to 18.1%) for attributable deaths, and 20.3% for YLLs (1362.9 YLLs; 95% UI, 1120.3 to 1829.5 YLLs) vs 1990, while YLD rates increased 9.8% (95% UI, 4.6% to 16.6%).

### Sex Ratios

By sex, 2021 age-adjusted rates of nervous system–related disorders showed a female to male ratio (FMR) of 1.1 (95% UI, 1.1 to 1.2). DALY rates were nearly identical (FMR, 1.0; 95% UI, 0.9 to 1.1). Deaths and YLL rates were greater for males (deaths: FMR, 0.9; 95% UI, 0.9 to 1.0; YLLs: FMR, 0.9; 95% UI, 0.8 to 0.9).

### Condition-Specific Temporal and Sex-Related Trends

Parkinson disease had the largest increase from 1990 in age-standardized rates of DALYs (43.5%; 95% UI, 38.3% to 47.5%), YLDs (25.7%; 95% UI, 10.0% to 41.3%), and YLLs (47.5%; 95% UI, 43.8% to 50.7%), while meningitis had the largest declines (DALYs: −75.5%; 95% UI, −76.9% to −74.1%; YLDs: −80.2%; 95% UI, −81.7% to 78.5%; YLLs: −75.2%; 95% UI, −76.7% to −73.7%).

Among neurodevelopmental disorders, age-standardized DALY rates of attention-deficit/hyperactivity disorder (ADHD) experienced the largest increase from 1990 at 10.4% (95% UI, 0.9% to 20.0%). Age-standardized DALY rates of idiopathic intellectual disability decreased (−13.3%; 95% UI, −20.4% to −9.5%).

For metabolic, infectious, congenital, and neonatal conditions affecting the nervous system, diabetic neuropathy had the steepest increase (151.8%; 95% UI, 123.4% to 182.0%) in the age-standardized DALY rate from 1990, while neural tube defects had the sharpest decrease in the same metric (−40.9%; 95% UI, −54.4% to −29.6%). Age-standardized death rates of neonatal encephalopathy from birth trauma or asphyxia and of neural tube defects decreased from 1990 by 33.7% (95% UI, 25.4% to 40.9%) and 43.8% (95% UI, 32.9% to 57.1%), respectively. The greater survivability of neural tube defects in 2021 led to large increases in prevalence (66.2%; 95% UI, 37.0% to 95.0%) and YLDs (65.4%; 95% UI, 36.4% to 94.8%) compared with 1990.

By condition in 2021, female sex predominance was most evidenced in age-standardized rates of migraine (DALYs: FMR, 2.1; 95% UI, 1.4 to 2.5) and multiple sclerosis (prevalence: FMR, 2.5; 95% UI, 2.4 to 2.6; deaths: FMR, 1.6; 95% UI, 1.5 to 1.7; YLDs: FMR, 2.4; 95% UI, 2.3 to 2.5; YLLs: FMR, 1.6; 95% UI, 1.5 to 1.7). Nervous system health loss among males was notable in age-standardized DALY rates for Parkinson disease (FMR, 0.5; 95% UI, 0.4 to 0.5), ADHD (FMR, 0.4; 95% UI, 0.3 to 0.4), autism spectrum disorder (FMR, 0.5; 95% UI, 0.4 to 0.5), congenital birth defects (FMR, 0.6; 95% UI, 0.4 to 0.8), and neurological consequences of syphilis (FMR, 0.2; 95% UI, 0.1 to 0.3).

Causes of nervous system health loss in 2021 varied along a continuum by age (Table 2). In children younger than 5 years, DALY rates per 100 000 persons were highest for neonatal encephalopathy due to birth asphyxia and injury (716.1; 95% UI, 641.1 to 795.1), autism spectrum disorder (230.7; 95% UI, 159.9 to 323.0), and preterm birth (192.3; 95% UI, 134.8 to 252.9). Older children and early adults (aged 5-19 years) had higher DALY rates per 100 000 persons from migraine (428.2; 95% UI, 28.1 to 1035.0), autism spectrum disorder (222.8; 95% UI, 154.7 to 312.4), and preterm birth (189.1; 95% UI, 133.9 to 248.9). Adults aged 20 to 59 years had the largest DALY rate per 100 000 persons from migraine (887.8; 95% UI, 137.1 to 1911.4), stroke (471.0; 95% UI, 432.8 to 512.6), and diabetic neuropathy (332.5; 95% UI, 222.5 to 468.4). For adults aged 60 to 79 years, DALY rates per 100 000 persons were greatest for stroke (2616.8; 95% UI, 2429.5 to 2806.0), diabetic neuropathy (1986.0; 95% UI, 1311.5 to 2741.8), and Alzheimer disease and other dementias (1753.2; 95% UI, 859.8 to 3891.6). Ranking DALY rates per 100 000 persons among US individuals aged 80 years or older, Alzheimer disease and other dementias led (16 014.6; 95% UI, 7137.2 to 33 736.9), followed by stroke (10 472.3; 95% UI, 8513.0 to 11 630.5) and diabetic neuropathy (2357.9; 95% UI, 1650.7 to 3250.3).

**Table 2.** Disability-Adjusted Life-Years (DALYs) per 100 000 People by 5 Broad Age Categories for All Conditions With Neurological Health Loss in the United States in 2021

Condition	Aged <5 y		Aged 5-19 y		Aged 20-59 y		Aged 60-79 y		Aged ≥80 y	
	DALYs (95% UI) per 100 000 persons	Rank	DALYs (95% UI) per 100 000 persons	Rank	DALYs (95% UI) per 100 000 persons	Rank	DALYs (95% UI) per 100 000 persons	Rank	DALYs (95% UI) per 100 000 persons	Rank
All neurological conditions	1739.4 (1552.7 to 1933.9)	NA	1357.2 (847.5 to 2085.7)	NA	2991.7 (2089.3 to 4241.1)	NA	9116.5 (7328.5 to 11 070.3)	NA	32 928 (24 321.8 to 48 158.1)	NA
Alzheimer disease and other dementias	0 (0.0 to 0.0)	34	0 (0.0 to 0.0)	35	64.4 (33.0 to 141.8)	12	1753.2 (859.8 to 3891.6)	3	16 014.6 (7137.2 to 33 736.9)	1
Attention-deficit/hyperactivity disorder	4.9 (2.4 to 8.8)	17	56.9 (28.4 to 99.3)	6	16 (8.6 to 25.9)	19	1 (0.4 to 1.8)	27	0 (0.0 to 0.0)	33
Autism spectrum disorder	230.7 (159.9 to 323.0)	2	222.8 (154.7 to 312.4)	2	201.6 (139.5 to 281.2)	4	150.4 (103.8 to 208.0)	11	72.6 (49.8 to 102.4)	14
Congenital birth defects	11.6 (6.5 to 19.3)	14	6.2 (2.2 to 12.9)	18	5.9 (2.1 to 12.5)	23	5.7 (2.0 to 12.2)	22	5.4 (1.9 to 11.6)	21
Congenital Zika syndrome	0 (0.0 to 0.0)	30	0 (0.0 to 0.0)	36	0 (0.0 to 0.0)	36	0 (0.0 to 0.0)	36	0 (0.0 to 0.0)	36
COVID-19	5.8 (0.4 to 24.5)	16	11.1 (0.7 to 50.1)	14	30.6 (0.8 to 101.3)	16	26.7 (0.8 to 78.9)	17	25.2 (0.8 to 68.4)	16
Cystic echinococcosis	0 (0.0 to 0.0)	29	0 (0.0 to 0.0)	33	0 (0.0 to 0.0)	34	0 (0.0 to 0.0)	34	0 (0.0 to 0.0)	31
Diabetes	0 (0.0 to 0.0)	28	1.3 (0.8 to 2.0)	25	332.5 (222.5 to 468.4)	3	1986 (1311.5 to 2741.8)	2	2357.9 (1650.7 to 3250.3)	3
Down syndrome	4.6 (3.0 to 6.5)	18	4.5 (3.0 to 6.4)	20	3 (2.0 to 4.2)	27	0.2 (0.1 to 0.3)	31	0 (0.0 to 0.0)	36
Encephalitis	23.8 (21.0 to 26.5)	11	4.5 (4.2 to 4.8)	21	5.5 (5.3 to 5.8)	25	11.3 (10.6 to 11.9)	20	21.3 (17.4 to 23.4)	17
Epilepsy	90.6 (57.4 to 143.3)	5	104.7 (58.1 to 176.4)	4	119.4 (77.6 to 178.6)	7	147.6 (88.6 to 242.4)	12	227.1 (136.9 to 377.9)	7
Fetal alcohol syndrome	1 (0.5 to 1.7)	22	1.1 (0.5 to 1.8)	26	0.7 (0.4 to 1.3)	30	0.4 (0.2 to 0.7)	29	0.4 (0.2 to 0.7)	25
Guillain-Barré syndrome	0.3 (0.2 to 0.5)	24	0.4 (0.2 to 0.7)	27	1.4 (0.9 to 2.0)	29	3.8 (2.3 to 5.7)	24	4.5 (2.6 to 6.8)	22
Idiopathic intellectual disability	20.3 (4.2 to 41.0)	12	25.7 (7.2 to 47.7)	11	18 (5.2 to 33.8)	18	9.2 (2.9 to 17.9)	21	6.9 (2.7 to 12.7)	20
Klinefelter syndrome	0.1 (0.0 to 0.2)	25	0 (0.0 to 0.1)	30	0 (0.0 to 0.0)	35	0 (0.0 to 0.0)	35	0 (0.0 to 0.0)	32
Meningitis	45.2 (40.2 to 50.7)	9	4.8 (4.5 to 5.0)	19	8.4 (8.1 to 8.8)	22	12.5 (11.8 to 13.1)	19	17.8 (14.4 to 19.6)	18
Migraine	0 (0.0 to 0.0)	36	428.2 (28.1 to 1035.0)	1	887.8 (137.1 to 1911.4)	1	453.1 (122.7 to 957.3)	5	224 (65.1 to 467.8)	9
Motor neuron disease	27.3 (24.5 to 30.2)	10	3.2 (2.9 to 3.6)	22	36.6 (35.2 to 38.1)	14	188.8 (177.1 to 197.0)	7	125.8 (102.9 to 138.5)	10
Multiple sclerosis	0 (0.0 to 0.0)	35	1.8 (1.2 to 2.4)	24	69.2 (57.8 to 81.0)	11	134.8 (120.8 to 147.9)	13	74.6 (63.0 to 84.2)	13
Neonatal encephalopathy	716.1 (641.1 to 795.1)	1	38.5 (28.1 to 51.1)	10	32.8 (23.9 to 43.5)	15	16.9 (11.9 to 22.1)	18	1.5 (1.0 to 2.1)	24
Neonatal jaundice	2.3 (1.6 to 3.0)	21	2.3 (1.7 to 3.1)	23	2.1 (1.5 to 2.8)	28	1.3 (0.9 to 1.7)	26	0.2 (0.2 to 0.3)	28
Neonatal sepsis	11.3 (6.7 to 16.5)	15	11.5 (6.9 to 16.7)	13	10.4 (6.2 to 15.1)	21	5.4 (3.2 to 7.9)	23	0.3 (0.2 to 0.5)	26
Nervous system cancer	68 (59.4 to 77.4)	6	69.2 (65.3 to 73.3)	5	144.6 (141.0 to 148.2)	5	401.1 (379.2 to 413.4)	6	313.7 (256.3 to 341.8)	5
Neural tube defects	157.5 (121.2 to 185.5)	4	6.3 (5.2 to 7.6)	17	5.8 (4.8 to 6.9)	24	2.3 (1.7 to 3.1)	25	1.8 (1.3 to 2.5)	23
Neurocysticercosis	0 (0.0 to 0.0)	27	0.3 (0.1 to 0.8)	28	22.3 (10.7 to 38.5)	17	62.6 (30.1 to 116.1)	15	98 (49.1 to 177.3)	12
Neurosyphilis	0 (0.0 to 0.0)	31	0 (0.0 to 0.0)	31	0.1 (0.1 to 0.2)	31	0.3 (0.2 to 0.4)	30	0.3 (0.2 to 0.5)	27
Other chromosomal anomalies	14.6 (9.5 to 21.0)	13	8.5 (5.6 to 12.0)	15	4.6 (3.1 to 6.4)	26	0.5 (0.3 to 0.7)	28	0 (0.0 to 0.0)	34
Other neurological disorders	56.5 (48.1 to 67.8)	7	48 (33.6 to 70.7)	7	76.5 (67.2 to 88.0)	10	186.2 (171.6 to 200.1)	8	283.8 (237.3 to 313.5)	6
Parkinson disease	0 (0.0 to 0.0)	33	0 (0.0 to 0.0)	34	15.2 (14.0 to 16.6)	20	468.9 (436.0 to 501.4)	4	2169.7 (1809.2 to 2363.5)	4
Preterm birth	192.3 (134.8 to 252.9)	3	189.1 (133.9 to 248.9)	3	143.7 (103.1 to 183.9)	6	59.2 (43.3 to 76.3)	16	13.6 (8.6 to 22.0)	19
Rabies	0.7 (0.6 to 0.8)	23	0.1 (0.1 to 0.1)	29	0 (0.0 to 0.0)	32	0 (0.0 to 0.0)	33	0.1 (0.1 to 0.2)	30
Spinal cord injury	3.5 (2.3 to 4.5)	19	16.6 (11.5 to 22.5)	12	100.7 (70.1 to 130.1)	9	167.6 (119.7 to 220.0)	9	117.1 (80.3 to 159.8)	11
Stroke	47.5 (42.5 to 52.7)	8	41 (33.1 to 49.4)	8	471 (432.8 to 512.6)	2	2616.8 (2429.5 to 2806.0)	1	10 472.3 (8513.0 to 11 630.5)	2
Tension-type headache	0 (0.0 to 0.0)	32	40.3 (5.3 to 200.3)	9	100.9 (27.5 to 349.6)	8	83.7 (24.8 to 273.6)	14	53 (12.2 to 190.2)	15
Tetanus	0.1 (0.0 to 0.1)	26	0 (0.0 to 0.0)	32	0 (0.0 to 0.0)	33	0.1 (0.0 to 0.1)	32	0.2 (0.1 to 0.2)	29
Traumatic brain injury	3 (2.0 to 4.2)	20	8.2 (5.6 to 11.5)	16	59.9 (41.8 to 80.7)	13	159 (113.0 to 210.9)	10	224.5 (156.4 to 294.7)	8

Abbreviations: NA, not applicable; UI, uncertainty interval.

### Nervous System Health by State

**Figure 3.**
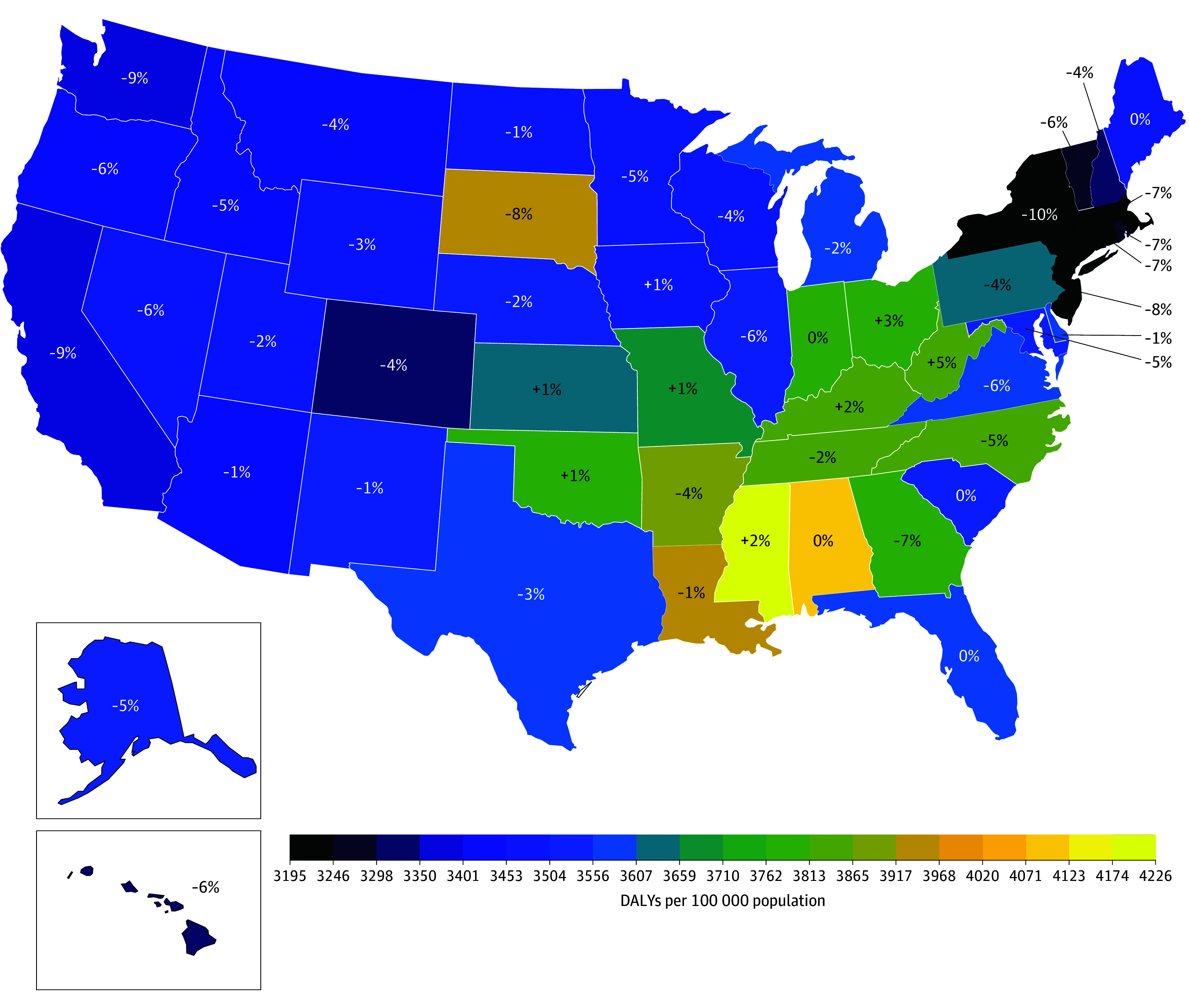
Rates of Age-Standardized Disability-Adjusted Life-Years (DALYs) per 100 000 Population for Disorders Affecting the Nervous System by State, 1990-2021 The color map depicts 2021 values. Percentages gained or lost refer to the difference between 2021 and 1990 DALYs.

Mississippi, Alabama, and Louisiana had the largest age-standardized DALY rate per 100 000 persons due to aggregated neurological health loss in 2021: 4225.8 (95% UI, 3316.4 to 5415.9), 4080.5 (95% UI, 3177.6 to 5138.0), and 3943.4 (95% UI, 3075.1 to 5022.1), respectively (Figure 3). Only 4 states (Mississippi, Ohio, Kentucky, and West Virginia) had more than a 1% increase in age-standardized DALYs per 100 000 persons in 2021 compared with 1990.

## Discussion

In 2021, the majority of US individuals experienced health loss due to neurological disorders, neurodevelopmental disorders, or a neurological consequence of metabolic, congenital, neonatal, or infectious conditions. Collective effects on US nervous system health are quantified in nearly half a million deaths and many millions of DALYs, YLLS, and YLDs.

Disorders affecting the nervous system accounted for an outsized share of disability, illness, and death in the United States in 2021. DALYs due to nervous system health loss were 13% of total 2021 US DALYs (16.6 million of 126.7 million DALYs)^21^ due to communicable and noncommunicable disease and injury, ahead of cardiovascular diseases (DALYs from stroke were not aggregated under cardiovascular diseases). Stroke and Alzheimer disease and other dementias were in the top 10 of 2021 noncommunicable diseases by DALYs and the top 5 causes of death in the United States.

US trends in conditions affecting the nervous system grew from 1990 to 2021 in total prevalence, deaths, YLLs, DALYs, and YLDs, but these metrics were mostly stable or declining in age-standardized rates. Growth in absolute nervous system health loss over 31 years is related to increases in the US population size (by 31.3%)^21^ and life expectancy (by 2.0%),^22,23^ with distribution of relevant conditions in older age groups.^11,21^ Standardizing health metrics by age and rates per unit population showed meaningful declines except in YLDs, as more disabling conditions preferentially affect an older population that is living longer. Declining age-standardized YLL and DALY rates coincide with widespread implementation of new treatments, including effective therapies for migraine,^24,25^ and ischemic stroke.^26^ Risk factor reduction, including smoking cessation, blood pressure control, and diabetes management, reduced the frequency and severity of stroke,^27,28^ the largest cause of US DALYs.

The greatest age-standardized DALY rates were in the southeastern United States, corresponding roughly to the Stroke Belt, defined by an age-adjusted mortality rate due to stroke more than 10% greater than that of the US national rate.^29^ These states are more rural and have a higher proportion of Black residents, greater rates of preventable risk factors, and more socioeconomically disadvantaged residents.^30^

Compared with the world at large, the US prevalence of conditions affecting the nervous system in 2021 is higher (54.2% of the US population vs 43.1% of the world population). The US age-standardized prevalence rate is the highest in the world, followed by this rate in Belgium and Norway. Attributable death rates from diseases affecting the nervous system were greater than the global figure at 143.7 deaths per 100 000 persons in the United States and 140.8 deaths per 100 000 persons globally, but substantially less when adjusting for age (75.8 deaths/100 000 persons vs 139.0 deaths/100 000 persons, respectively). Unadjusted DALY rates per 100 000 population due to loss of neurological health were greater globally than in the United States (5621.9 vs 4960.9 DALYs/100 000 persons), but the age-standardized DALY rate per 100 000 persons was markedly less in the United States than worldwide (3546.9 vs 5673.6 DALYs/100 000 persons, respectively). Total DALYs increased 55.2% in the United States from 1990 to 2021, with only an 18.2% global increase over the same period. Age-standardized DALY rates for stroke, Alzheimer disease and other dementias, migraine, and diabetic neuropathy ranked in the top 5 globally and in the United States. These figures are consistent with an older US population more vulnerable to age-associated disabling and fatal conditions affecting the nervous system than that worldwide.^21^

Ranking by age-standardized rates among 64 other high-income countries and territories,^10,31^ the United States was 47th in YLDs (between Hungary and Oman), 33rd in deaths (between the Czech Republic and the Netherlands), 36th in YLLs (between Chile and Estonia), and 37th in DALYs (between the Czech Republic and Greece). This places the United States firmly in the middle rank of nations with similar incomes in loss of nervous system health.

This study is an analytic subsample of the global burden of disorders affecting the nervous system for 2021,^10^ and it updates prior estimates of the US burden of neurological disorders.^13,14^ We used identical methods and 36 of 37 conditions cited in the global analysis, which adds neurodevelopmental, congenital, neonatal, and systemic disorders with nervous system consequences for a broader examination of nervous system health loss than previous analyses. Gooch et al^14^ estimated that 100 million US individuals in 2011 had a neurological disease, and they extracted the direct costs of 8 common neurological disorders from published literature. Feigin et al^13^ followed GBD methods using published sources, administrative data, and modeling, dropping low back pain and adding another 7 conditions to better represent neurological disorders affecting the United States in terms of disability and deaths. The current analysis is broader yet, with the addition of 21 disorders causing nervous system health loss.

### Limitations

This study has limitations. First, we use a GBD framework to assess DALYs, deaths, YLLs, and YLDs, not fiscal costs associated with these disorders. Second, we did not detail health loss from every neurological disorder. Some conditions, such as Alzheimer disease and other dementias, were aggregated to maintain consistency with the GBD framework. An umbrella category of residual neurological disorders ranked 6th to 11th in prevalence in the 5 age bins, suggesting that condition-specific analyses within this category may be warranted. Some multisystem diseases (eg, HIV) were excluded because the neurological component could not be easily extricated. Third, the sheer volume of conditions and findings risks overshadowing some important disorders in favor of the most prevalent or most attributable DALYs. Multiple sclerosis became more prevalent and had increases in mortality and disability from 1990 to 2021 in the United States, but it represented a small fraction in each measure. Fourth, mortality is excluded for some conditions. Deaths due to injuries in GBD are assigned to the cause of the injury (eg, spinal injury and motor vehicle collision); mortality was not a direct function of the disease (neurodevelopmental disorders), associated explicitly with the disease (migraine, tension-type headache), or a nervous system consequence of the disease (eg, diabetic neuropathy). Fifth, GBD trends are evaluated over decades, not annually, so a year-by-year analysis to selectively determine the effects of COVID-19^32^ or diminished life expectancy in 2020 through 2021^33,34^ were outside of the scope of this study.

## Conclusions

The findings of this cross-sectional study of the Global Burden of Disease 2021 study data represent a call to better address disorders affecting the nervous system among a growing and aging population. Noncommunicable disorders with neurological effects are a highly prevalent source of disability and mortality. Trends over time show that widespread implementation of effective treatments for conditions like stroke improve outcomes. Stability or growth over 3 decades in the burden of neurodevelopmental disorders (such as autism spectrum disorder), neurodegenerative diseases (like motor neuron disease and Parkinson disease), and neurological effects of systemic disorders (including diabetes) show a failure in development and/or dissemination of effective disease-modifying therapies.^35^ The burden of lost health is accentuated by reductions in mortality for disorders across the age spectrum, where more life-years equate to greater accumulated disability. National health priorities should encourage robust funding for bench research to better understand causes for these disorders, translational studies to take work from the bench to the bedside, clinical trials in preparation for effective treatments, and postapproval investigations of comparative effectiveness, dissemination, and implementation.^36,37,38^ Streamlining drug approval processes, incentivizing evidence-based care, and improving access to neurological specialists and disability care could yield vast dividends in US national health in the next 30 years.^39,40,41,42,43,44^

## Notes

Supplement 1. **eTable 1.** Conditions Included in the Analysis of Nervous System Health Loss **eTable 2.** Neurological Features of Conditions That Impact Multiple Systems **eTable 3.** Case Definitions and Adjustments **eTable 4.** Conditions in Other Neurological Disorders Category **eTable 5.** Input Data to Nonfatal Models and Most Recent Dates for Data Seeking **eTable 6.** Source Counts in Fatal Models

Supplement 2. Data Sharing Statement
