# Supplementary material for: US Burden of Disorders Affecting the Nervous System: From the Global Burden of Disease 2021 Study
Source: JAMA Neurol. 2025 Nov 24;83(1):20–34. doi: 10.1001/jamaneurol.2025.4470 (PMC12645399; doi:10.1001/jamaneurol.2025.4470)
Supplement: Supplement 1. — eTable 1. Conditions Included in the Analysis of Nervous System Health Loss eTable 2. Neurological Features of Conditions That Impact Multiple Systems eTable 3. Case Definitions and Adjustments eTable 4. Conditions in Other Neurological Disorders Category eTable 5. Input Data to Nonfatal Models and Most Recent Dates for Data Seeking eTable 6. Source Counts in Fatal Models [file jamaneurol-e254470-s001.pdf]

Supplemental Online Content

Ney JP, Steinmetz JD, Anderson-benge E, et al. US burden of disorders affecting the nervous system: from the Global Burden of Disease 2021 study. *JAMA Neurol*. Published online November 24, 2025. doi:10.1001/jamaneurol.2025.4470

eTable 1. Conditions Included in the Analysis of Nervous System Health Loss

eTable 2. Neurological Features of Conditions That Impact Multiple Systems

eTable 3. Case Definitions and Adjustments

eTable 4. Conditions in Other Neurological Disorders Category

eTable 5. Input Data to Nonfatal Models and Most Recent Dates for Data Seeking

eTable 6. Source Counts in Fatal Models

This supplemental material has been provided by the authors to give readers additional information about their work.

© 2025 Ney JP et al. *JAMA Neurology*.

eTable 1. Conditions Included in the Analysis of Nervous System Health Loss

Columns indicate whether conditions are entirely neurological (full contribution) versus those where only components of the condition contribute to nervous system health loss (partial contribution) and whether conditions are included in the mortality analyses reported here. Guillain-Barré syndrome deaths not ascribed to an underlying cause are captured under "Other neurological disorders."

Conditions included in current analysis	Contribution to nervous system health loss	Mortality included in analysis
Alzheimer's disease and other dementias	Full	X
Attention-deficit/hyperactivity disorder	Full	
Autism spectrum disorders	Full	
Encephalitis	Full	X
Epilepsy	Full	X
Fetal alcohol syndrome	Full	
Guillain-Barré syndrome (GBS)	Full	X
Idiopathic developmental intellectual disability	Full	
Meningitis	Full	X
Migraine	Full	
Motor neuron disease	Full	X
Multiple sclerosis	Full	X
Neonatal encephalopathy	Full	X
Neural tube defects	Full	X
Nervous system cancers (CNS; neuroblastoma and other peripheral nervous cell tumours)	Full	X
Neurocysticercosis	Full	X
Other neurological disorders (including degenerative diseases, disorders of the autonomic nervous system, movement disorders, spinocerebellar disease, nerve root and plexus disorders, peripheral nerve disorders, neuromuscular disorders and muscle diseases such as myopathies)	Full	X
Parkinson's disease	Full	X
Rabies	Full	X
Spinal cord injury	Full	
Stroke (ischaemic stroke, subarachnoid haemorrhage, intracerebral haemorrhage)	Full	X
Tension-type headache	Full	
Tetanus	Full	

© 2025 Ney JP et al. *JAMA Neurology*.

Traumatic brain injury	Full	
Congenital birth defect	Partial	
COVID-19 (cognitive impairment and GBS)	Partial	
Diabetes (diabetic neuropathy)	Partial	
Down syndrome	Partial	
Echinococcosis (cystic)	Partial	
Haemolytic disease and other neonatal jaundice (neonatal jaundice)	Partial	
Klinefelter syndrome	Partial	
Neonatal sepsis	Partial	
Other chromosomal abnormalities	Partial	
Preterm birth	Partial (all sequelae)	
Syphilis (congenital and adult neurosyphilis)	Partial	
Zika virus disease (congenital)	Partial	

© 2025 Ney JP et al. *JAMA Neurology*.

eTable 2. Neurological Features of Conditions That Impact Multiple Systems

**Condition**	**Neurological features**
Congenital birth defects	Cognitive impairment, motor impairment, intellectual disability, hearing loss
COVID-19	Cognitive impairment, Guillain-Barré syndrome (due to COVID)
Diabetes	Diabetic neuropathy (including diabetic foot and amputation)
Down syndrome	Intellectual disability, dementia
Echinococcosis	Epilepsy
Klinefelter syndrome	Intellectual disability
Neonatal jaundice	Long-term consequences: motor impairment, cognitive impairment, epilepsy, blindness
Neonatal sepsis	Long-term consequences: motor impairment, cognitive impairment, epilepsy, blindness
Neurocysticercosis	Epilepsy
Other chromosomal abnormalities	Intellectual disability, dementia
Preterm birth	Long-term consequences: motor impairment, cognitive impairment, epilepsy, blindness, retinopathy of prematurity
Syphilis	Adult and congenital neurosyphilis
Zika virus disease	Congenital (microcephaly)

© 2025 Ney JP et al. *JAMA Neurology*.

eTable 3. Case Definitions and Adjustments

Non-reference case definition data were adjusted to reference. Input data using different case definitions were matched by age, sex, location, and year; where possible, within-study comparisons or validation studies were used. Matched pairs were logit-transformed, and the logit difference was calculated and used as input data into a meta-regression tool called MR-BRT (meta-regression—Bayesian, regularised, trimmed). Meta-regression results were used to systematically adjust non-reference data up or down to account for under- or over-counting, respectively.

**Condition**		**Definition**
ADHD	Reference	An externalising disorder characterised by persistent inattention and/or hyperactivity-impulsivity. As per criteria set by the Diagnostic and Statistical Manual of Mental Disorders (DSM) Fourth Edition, Text Revision, diagnosis requires 6+ symptoms of inattention or hyperactivity-impulsivity to have persisted for at least six months in two or more settings causing significant impairment to functioning, with at least some impairing symptoms present prior to 7 years of age (12 years of age in DSM-5).
Alzheimer's disease and other dementias	Reference	A progressive, degenerative, and chronic neurological disorder typified by memory impairment and other neurological dysfunctions. Defined based on DSM III, IV or V, or ICD case criteria, including cognitive deficits that must include memory impairment, functional impairment, and gradual onset and continued decline.
	Alternatives	Diagnosis from clinical records, algorithm criteria, National Institute on Aging Alzheimer's disease criteria, 10/66 algorithm criteria, general practitioner records.
Autism spectrum disorder	Reference	A group of neurodevelopmental disorders with onset occurring in early childhood, characterised by pervasive impairment in several areas of development, including social interaction and communication skills, along with restricted and repetitive patterns of behaviours and/or interests, as defined by DSM-5 criteria or equivalent in International Classification of Diseases (ICD) or Chinese Classification of Mental Disorders (CCMD), and estimated from general population

© 2025 Ney JP et al. *JAMA Neurology*.

Condition		Definition
		surveys with additional case-finding or total population screening.
	Alternatives	Study captures autism instead of autism spectrum disorder, study is a general population survey without additional case finding.
Congenital birth defects	Reference	For this analysis, this category includes congenital heart defects, other congenital anomalies, Edwards syndrome and Patau syndrome, and other chromosomal abnormalities that have neurological outcomes. The case definition of congenital anomalies includes any condition present at birth that is a result of abnormalities of embryonic development, excluding those directly the result of infections or substance abuse, and excludes minor anomalies as defined by European Surveillance of Congenital Anomalies (EUROCAT). Registries with the most complete list of reported case definitions (highest case ascertainment) were used as reference registries for each condition in the congenital birth defect category.
	Alternatives	Registries that did not have the highest level of case ascertainment.
Congenital Zika virus syndrome	Reference	Zika virus infection during pregnancy leading to fetal neurological symptoms such as microcephaly or decreased brain volume, and other congenital malformations. Cases identified from official reports primarily from the Pan American Health Organization (PAHO), which further describes criteria for maternal exposure and child phenotypes.[1]
COVID-19 (long)	Reference	A SARS-CoV-19 infection leading to new and persistent cognitive impairment that impacts everyday functioning and lasts at least three months after acute infection symptom onset.
	Alternatives	Outcome definition of cognitive impairment based on “memory problems”.
Cystic echinococcosis	Reference	A parasitic disease caused by infection with the *Echinococcus granulosis* tapeworm that can spread to humans through ingestion of soil, water, or food contaminated with the faecal matter of an infected dog containing infective eggs. Diagnosis

© 2025 Ney JP et al. *JAMA Neurology*.

Condition		Definition
		comes from clinical findings, imaging, serology, and tissue pathology.
Diabetic neuropathy	Reference	Diabetes: A chronic condition where either the pancreas does not produce enough insulin or the body is not able to metabolise insulin properly. This is defined as a fasting plasma glucose concentration ≥ to 7mmol/L, or use of insulin or diabetes medication. Neuropathy: People with diabetes mellitus who have diabetic neuropathy determined by microfilament test. Diabetic foot: People with diabetes mellitus who have diabetes foot, which is a poorly healing ulcer. Amputation: People with diabetes mellitus who have a lower limb amputation.
	Alternatives	Diabetes: Blood glucose tests other than reference (includes fasting plasma glucose threshold other than 7mmol/L, post-prandial glucose test, oral glucose tolerance test, glycated haemoglobin, and various cutoffs for each test) or USA and Taiwan insurance claims data. Neuropathy: Diagnosis determined with test other than microfilament. Amputation: Amputation of specific part of lower limb (e.g., toes only, feet only, below ankle only).
Down syndrome	Reference	Also known as Trisomy 21, is the presence of a third copy of chromosome 21, typically caused by nondisjunction during the production of gametes. Down syndrome is associated with several specific physical characteristics, including decreased muscle tone, flat facial features, an upward slant to the eyes, abnormally shaped ears, a single deep crease across the centre of the palm, folded skin on the inner corners of the eyes, and ability to extend joints beyond the usual, among others.
Encephalitis	Reference	A disease caused by acute inflammation of the brain, which can cause flu-like symptoms such as headaches, fever, drowsiness, fatigue, and at times, seizures, hallucinations, or stroke. Reference definition is based on ICD-10 criteria from inpatient data.

© 2025 Ney JP et al. *JAMA Neurology*.

Condition		Definition
	Alternatives	Diagnosis from USA private claims data or epidemiological surveillance.
Epilepsy	Reference	A condition characterised by recurrent (two or more) epileptic seizures, unprovoked by any immediate identified cause. Active epilepsy is at least one epileptic seizure in the past five years, regardless of antiepileptic drug treatment. Diagnosis criteria based on “Guidelines for Epidemiological Studies on Epilepsy” (commissioned by the International League Against Epilepsy).
	Alternatives	Lifetime recall of epilepsy.
Fetal alcohol syndrome	Reference	Caused by maternal drinking during pregnancy and the most severe form of fetal alcohol spectrum disorder (FASD); other forms of FASD including partial fetal alcohol syndrome, alcohol-related neurodevelopmental disorder, and alcohol-related birth defects are not included. The syndrome is characterised by certain patterns of facial anomalies, growth retardation, and central nervous system neurodevelopmental abnormalities. Diagnostic criteria for active case finding comes from the USA Institute of Medicine, the British Paediatric Association, and other recognised bodies in the area.
	Alternatives	Passive case finding
Guillain-Barré syndrome	Reference	An immune-mediated nerve dysfunction that usually occurs as a complication of respiratory or gastrointestinal infection and leads to rapid onset of weakness in the feet and legs, and sometimes the arms, which then progresses toward the trunk. Cases are identified by doctor diagnosis or other record, including ICD-coded claims or hospital data.
Idiopathic intellectual disability	Reference	A condition of below-average mental ability originating before age 18, as defined by the American Association on Intellectual and Developmental Disabilities. A prevalent case is defined as an IQ score <70.
Klinefelter syndrome	Reference	Also known as 47 XXY, is a condition in which a male is born with an extra X chromosome in all or some of his cells; here the definition also includes

© 2025 Ney JP et al. *JAMA Neurology*.

Condition		Definition
		other genotypes with supernumerary X chromosomes, eg, XXXY, XXXXY, etc. The primary feature is sterility, but it can cause a variety of other conditions, including weaker muscles, increased height, poor coordination abilities, smaller genitals, breast growth, and reduced sexual drive as a result of lower testosterone levels.
Meningitis	Reference	A disease caused by inflammation of the meninges, the protective membrane surrounding the brain and spinal cord, that is typically caused by an infection in the cerebrospinal fluid. Symptoms include headache, fever, stiff neck, and sometimes seizure. Gold-standard diagnosis in inpatient hospital clinical data or literature via antigen test, blood test, cerebrospinal fluid test, or latex agglutination test.
	Alternatives	Diagnosis from private insurance claims data or via epidemiological surveillance.
Migraine		A disabling primary headache disorder, typically characterised by recurrent moderate or severe unilateral pulsatile headaches, either without aura or with aura (transient neurological symptoms). Diagnosis based on International Classification of Headaches (ICD-3) criteria of 5+ attacks that (1) last 4–72 hours, (2) causes nausea and/or vomiting or photophobia and phonophobia, (3) has at least two of the following – unilateral location, pulsating quality, moderate or severe pain, aggravation by or causing avoidance of routine physical activity; (4) not due to other diagnosis. Definite migraines meet all of the above criteria and probable meet all but one of the above criteria.
	Alternatives	Other than one-year recall, not representative study population, low-quality methods (sampling, survey, diagnostic instrument, diagnostic criteria), poor response rate, headache type assumed
Motor neuron disease	Reference	A set of chronic, degenerative, and progressive neurological conditions typified by the destruction of motor neurons and the subsequent deterioration of voluntary muscle activity. The

© 2025 Ney JP et al. *JAMA Neurology*.

Condition		Definition
		most common type is amyotrophic lateral sclerosis (ALS). Gold-standard diagnosis uses the El Escorial Criteria with clinical examination, as well as imaging and electrophysiology.
	Alternatives	Surveys limited to ALS case finding only.
Multiple sclerosis	Reference	A chronic, degenerative, and progressive condition typified by damage to the myelin sheaths around neurons. Accepted diagnostic criteria include McDonald's, Poser, Schumacher, and McAllen), or diagnosis via a clinical neurological exam.
Neonatal encephalopathy	Reference	Clinical diagnosis of disturbed neurological function in the earliest days of life in an infant born at or beyond 35 weeks' gestation, manifested by reduced level of consciousness or seizures, often accompanied by difficulty initiating and maintaining respiration, and by depression of tone and reflexes.
Neonatal jaundice	Reference	Clinical diagnosis of pathological neonatal jaundice or total serum bilirubin greater than expected for postnatal age and prematurity (hyperbilirubinaemia).
Neonatal preterm birth	Reference	Newborn born alive and less than 36 completed weeks of gestation; ie, birth between [0, 37.0) weeks of gestation; no adjustment is currently made between different forms of gestational age dating (obstetric estimate, last menstrual period, or other).
Neonatal sepsis	Reference	Includes infections during the neonatal period that advance to a systemic bloodstream infection (sepsis) and infections that occur during the neonatal period that are not already modelled separately in the GBD.
Nervous system cancers	Reference	Malignant neoplasm of the brain or central nervous system, or peripheral nervous system. Note: This combined nervous system cancer category is composed of two cancer groupings explicitly modelled in the GBD: "Brain and central nervous system cancer" and "Neuroblastoma and other peripheral nervous cell tumours". This category encompasses paediatric and adult cases, which includes primary malignancies but

© 2025 Ney JP et al. *JAMA Neurology*.

Condition		Definition
		not metastases. Although rare, PNS tumours are included in the category.
Neural tube defects	Reference	Includes anencephaly, which is the absence of a major portion of the brain, skull, and scalp, encephalocele, which is characterised by sac-like protrusions of the brain and meninges through openings in the skull, and spina bifida, which is when part of the spinal cord and/or meninges are uncovered by skin. The reference definitions refer to livebirths including those with chromosomal anomalies.
	Alternatives	Livebirths excluding chromosomal diagnoses, livebirths and stillbirths
Neurocysticercosis	Reference	A parasitic disease caused by the pig tapeworm *Taenia solium* that leads to cysts in the brain and epilepsy. Diagnosis is made in epilepsy patients with either *T. Solium* identified in excised cysticerci from tissues by microscopic examination or identification of cysticerci using magnetic resonance imaging (MRI), computerised tomography (CT), or X-ray brain scans to identify cysts and a positive result on CDC immunoblot assay.
	Alternatives	An epilepsy patient with calcified cystic lesions in the brain identified by CT scan, MRI, or X-ray, or a positive result on CDC immunoblot assay ("probable" case).
Other chromosomal abnormalities	Reference	Unbalanced chromosomal rearrangements, which are genetic anomalies that typically occur due to meiotic non-disjunction, when homologous chromosomes do not separate normally in nuclear division during gamete formation. Other chromosomal arrangements included here are Triple X syndrome, other meiotic non-disjunction events, other female sex chromosome abnormalities, and other unspecified chromosomal abnormalities.
Other neurological disorders	Reference	A residual category which groups together neurological conditions that are not directly estimated in the GBD, for example muscular dystrophy, Huntington disease, and myasthenia

© 2025 Ney JP et al. *JAMA Neurology*.

Condition		Definition
		gravis. A full list of included causes included in this category are listed in Supplemental Table 4.
Parkinson's disease	Reference	A chronic, degenerative, and progressive neurological condition typified by loss of motor mobility and control, most notably causing tremors. Requires the presence of two of the four primary symptoms and is consistent with the Gelb criteria: (1) tremors/trembling, (2) bradykinesia, (3) stiffness of limbs and torso, and (4) posture instability.
	Alternatives	Not population-representative, study excludes nursing homes, study uses the Movement Disorder Society (MDS) criteria, United Kingdom Parkinson's Disease Society (UKPD) Brain Bank criteria, or does not use explicit criteria.
Rabies	Reference	A viral infection transmitted by animal bite that is almost universally fatal without prophylactic vaccination. Clinical diagnosis of rabies excludes cases where intervention prevented disease following animal bite.
Spinal cord injury	Reference	A spinal cord lesion at or below the cervical level that leads to partial or total paralysis depending on the level and degree of injury.
Stroke (intracerebral haemorrhage)	Reference	A focal collection of blood within the brain parenchyma or ventricular system that is not caused by trauma. Stroke cases are considered acute from the day of incidence of a first-ever stroke through day 28 following the event. Stroke cases are considered chronic beginning 28 days following the occurrence of an event; includes all recurrent stroke events.
	Alternatives	Sources including first and recurrent strokes, estimates reported for all subtypes combined, estimates reported only for cases which survived to hospital admission.
Stroke (ischaemic)	Reference	Neurological dysfunction caused by focal cerebral, spinal, or retinal infarction. Stroke cases are considered acute from the day of incidence of a first-ever stroke through day 28 following the event. Stroke cases are considered chronic beginning 28 days following the occurrence of an event, and include all recurrent stroke events.

© 2025 Ney JP et al. *JAMA Neurology*.

Condition		Definition
	Alternatives	Sources including first and recurrent strokes, estimates reported for all subtypes combined, estimates reported only for cases which survived to hospital admission.
Stroke (subarachnoid haemorrhage)	Reference	Bleeding into the subarachnoid space (the space between the arachnoid membrane and the pia mater of the brain or spinal cord). Stroke cases are considered acute from the day of incidence of a first-ever stroke through day 28 following the event. Stroke cases are considered chronic beginning 28 days following the occurrence of an event, and include all recurrent stroke events.
	Alternatives	Sources including first and recurrent strokes, estimates reported only for aneurysmal subarachnoid haemorrhage, estimates reported only for cases which survived to hospital admission.
Syphilis	Reference	Infection with the *Treponema pallidum* bacterium usually spread by sexual contact or from a pregnant person to offspring; we account here for acute and chronic infection, with or without symptoms, and sequelae of congenital cases that persist after treatment.
	Alternatives	Private insurance claims.
Tension-type headache		Characterised by a dull, non-pulsatile, diffuse, band- or vice-like pain of mild to moderate intensity in the head or neck. Diagnosed based on ICD-3 criteria of 10+ attacks that (1) last 30 minutes to 7 days, (2) no nausea or vomiting and no more than one of photophobia or phonophobia, (3) has at least two of the following – bilateral location, pressing or tightening quality, mild or moderate pain, not aggravated by routine physical activity, (4) not due to other diagnosis. Definite migraines meet all of the above criteria, and probable meet all but one of the above criteria.
	Alternatives	Other than one-year recall, not representative study population, low-quality methods (sampling, survey, diagnostic instrument, diagnostic criteria), poor response rate, headache type assumed

© 2025 Ney JP et al. *JAMA Neurology*.

Condition		Definition
Tetanus	Reference	A life-threatening disease caused by infection with the toxin-producing bacterium *Clostridium tetani* and acquired via contamination of wounds. Tetanus is typically characterised by generalised, painful muscular spasms, with complications including mechanical respiratory failure, autonomic dysfunction, and death. Neonatal tetanus is often caused by contamination of the umbilical stump; initial symptoms include failure to feed and excessive crying, progressing to the typical clinical presentation of tetanus.
Traumatic brain injury	Reference	Injury to the head that causes short-term and in some cases long-term damage to the brain, manifesting in loss of concentration, headaches, memory problems, nausea, dizziness, and/or mood changes.

© 2025 Ney JP et al. *JAMA Neurology*.

eTable 4. Conditions in Other Neurological Disorders Category

Broad category		Conditions*
Nervous system		ICD 9: other and unspecified disorders of the nervous system;
Brain		ICD-9: cerebral degenerations usually manifest in childhood; idiopathic normal pressure hydrocephalus; corticobasal degeneration; cerebral degeneration in diseases classified elsewhere; other cerebral degeneration or unspecified; other demyelinating diseases of central nervous system<br>ICD-10: other demyelinating diseases of central nervous system; dementia in other diseases classified elsewhere; other degenerative diseases of basal ganglia; systemic atrophies primarily affecting central nervous system in diseases classified elsewhere
Movement disorders		ICD-10: Huntington's disease; other extrapyramidal and movement disorders; extrapyramidal and movement disorders in diseases classified elsewhere; other extrapyramidal disease and abnormal movement disorders; dystonia;
Spinocerebellar disease		ICD-9: spinocerebellar disease<br>ICD-10: hereditary ataxia
Diseases of the spinal cord		ICD-9: other diseases of spinal cord<br>ICD-10: other and unspecified diseases of spinal cord; spinal muscular atrophy and related syndromes
Autonomic nervous system		ICD-9: disorders of the autonomic nervous system<br>ICD-10: disorders of the autonomic nervous system
Nerve root/plexus disorders		ICD-9: other or unspecified nerve root and plexus disorders

© 2025 Ney JP et al. *JAMA Neurology*.

Peripheral nerve disorders**		ICD-9: acute infective polyneuritis; polyneuropathy; hereditary and idiopathic peripheral neuropathy<br>ICD-10: inflammatory polyneuropathy
Neuromuscular disorders		ICD-9: myoneural disorders (eg, myasthenia gravis, Lambert-Eaton syndrome); neonatal myasthenia gravis<br>ICD-10: myasthenia gravis and other myoneural disorders; disorders of myoneural junction and muscle in diseases classified elsewhere
Muscle diseases		ICD-9: muscular dystrophies and other myopathies<br>ICD-10: primary disorders of muscles, dermatomyositis, other and unspecified myopathies

*Listed conditions correspond to three-digit ICD 9 and 10 coding unless four-digit is required to code to an included neurological category.
**This condition category includes fatal outcomes of Guillain-Barré syndrome.

© 2025 Ney JP et al. *JAMA Neurology*.

eTable 5. Input Data to Nonfatal Models and Most Recent Dates for Data Seeking

Condition*	Prevalence	Incidence	Other	Countries with data	# of sources from U.S.^
ADHD	172	2	17	49	30
Alzheimer's disease and other dementias	254	92	225	58	85
Autism spectrum disorders	105	0	6	31	2
Congenital birth defects	1746	0	188	105	
COVID-19 (long-term)	0	0	26	14	5
Diabetic neuropathy (not total diabetes)	0	0	71		3
Down syndrome	1510	0	40	78	126
Echinococcosis	0	358	0	62	31
Encephalitis	0	392	1	57	51
Epilepsy	384	89	188	94	50
Fetal alcohol syndrome	169	0	8	32	29
Guillain-Barré syndrome	0	357	44	47	62
Headache disorders (migraine)	148	4	7	51	11
Headache disorders (tension-type)	94	0	6	39	1
Intellectual disability	64	0	0	31	9
Klinefelter syndrome	834	0	3	45	46
Meningitis	0	556	345	96	222
Motor neuron disease	24	48	1	18	28
Multiple sclerosis	211	86	29	53	37
Neonatal encephalopathy	331	0	747	65	88
Neonatal jaundice	62	1	1240	191	2
Neonatal sepsis	0	0	349	57	47
Nervous system cancers**	6	6230	641	101	475
Neural tube defects	1566	0	13	90	127
Neurocysticercosis	30	0	0	16	1

© 2025 Ney JP et al. *JAMA Neurology*.

Other chromosomal abnormalities	1330	0	23	71	109
Other neurological disorders	N/A (death data only for this group)				
Parkinson's disease	127	45	42	45	24
Preterm birth	0	0	2187	176	82
Rabies	N/A (death data only)				
Spinal cord injury	N/A (nature of injury is calculated from causes of injury)				
Stroke (ischaemic)	145	351	173	78	88
Stroke (intracerebral haemorrhage)	143	359	144	76	89
Stroke (subarachnoid haemorrhage)	131	288	102	63	85
Syphilis	996	667	24	177	99
Tetanus	0	0	258	50	73
Traumatic brain injury	N/A (nature of injury is calculated from causes of injury)				
Zika virus disease (congenital)	0	247	7	60	9

*Source counts are for the entire condition and not just the neurological component unless otherwise specified; **nervous system cancers refers to the combination of "Brain and central nervous system cancer" and "Neuroblastoma and other peripheral nervous cell tumours"; ^additive across measures. https://ghdx.healthdata.org/gbd-2021/sources

© 2025 Ney JP et al. *JAMA Neurology*.

eTable 6. Source Counts in Fatal Models

Condition	Vital registration and other death data	Number of countries	# of sources from the U.S.
Alzheimer's disease and other dementias†	Does not use vital registration or other death data		
Encephalitis	3719	147	40 sources of vital registration data and 39 sources of administrative data available for U.S. analyses
Idiopathic epilepsy	3560	148	
Meningitis	4090	163	
Motor neuron disease	3398	124	
Multiple sclerosis	3634	125	
Neonatal encephalopathy	3085	154	
Nervous system cancers*	6347	160	
Neural tube defects	3161	151	
Neurocysticercosis	3354	122	
Other neurological disorders	2823	127	
Parkinson's disease	3437	129	
Rabies	3700	147	
Stroke (any)	4017	152	
Tetanus	4075	160	

*Nervous system cancers refers to the combination of "Brain and central nervous system cancer" and "Neuroblastoma and other peripheral nervous cell tumours"; †data come from non-fatal modelling, which is used to inform fatal estimates.
https://ghdx.healthdata.org/gbd-2021/sources

© 2025 Ney JP et al. *JAMA Neurology*.
