# Supplementary material for: US Burden of Disorders Affecting the Nervous System: From the Global Burden of Disease 2021 Study
Source: JAMA Neurol. 2025 Nov 24;83(1):20–34. doi: 10.1001/jamaneurol.2025.4470 (PMC12645399; doi:10.1001/jamaneurol.2025.4470)
Supplement: Supplement 2. — Data Sharing Statement [file jamaneurol-e254470-s002.pdf]

Data Sharing Statement

Ney. US Burden of Disorders Affecting the Nervous System. *JAMA Neurol*. Published November 24, 2025. doi:10.1001/jamaneurol.2025.4470

Data
Data available: Yes
Data types: Data (not involving human participants), Data dictionary
How to access data: Flat files of all estimates included in paper
When available: With publication

Supporting Documents
Document types: None

Additional Information
Who can access the data: Anyone requesting
Types of analyses: any purpose
Mechanisms of data availability: public
Any additional restrictions: no
